# Predation, prey preference and switching behaviour of the soil predatory mite *Blattisocius mali* (Mesostigmata: Blattisociidae) on immature stages of *Drosophila hydei*

**DOI:** 10.1007/s10493-026-01187-y

**Published:** 2026-08-31

**Authors:** Muhammad Arslan Ibrahim, Katarzyna Michalska, Marcin Studnicki

**Affiliations:** 1 Department of Plant Protection, Warsaw University of Life Sciences, Nowoursynowska 159, Warsaw, 02-776 Poland; 2 Department of Biometry, Warsaw University of Life Sciences, Nowoursynowska 159, Warsaw, 02-776 Poland

**Keywords:** Soil predatory mite, Predatory behaviour, *Tyrophagus putrescentiae*, Diet, Oviposition, Survival

## Abstract

*Blattisocius mali* is a soil predatory mite known to disperse phoretically on adult drosophilid fruit flies and feed ectoparasitically on them during transport. Despite this association, its behavioural and trophic interactions with immature drosophilid stages remain poorly understood. This study analysed *B. mali*’s ability to prey on *Drosophila hydei* immature stages and examined the effects of prior rearing diet on predation, prey preference, and switching. Two types of predatory mite were compared: (1) fly-reared (FR) and (2) mite-reared (MR), raised on fruit flies or acarid mites, respectively. Predator females successfully attacked all developmental stages, except pupae, during 24 h of exposure in our assay. In non-choice tests, mean predation rates were highest on first-instar (L1), followed by eggs, second-instar (L2), and third-instar larvae (L3). FR predators, as compared to MR predators, showed the highest predation rates on all prey stages except L3. Feeding on fly eggs, *B. mali* exhibited significantly higher fecundity, laying nearly twice as many eggs compared to when they were fed on L1. Their oviposition declined markedly on L2 and L3. In two-choice and three-choice assays, *B. mali* showed a clear preference for eggs over larval instars in both predator types. FR predators preferred L1 over L2, while, conversely, MR predators preferred L2. Furthermore, *B. mali* females displayed different behaviour depending on the proportion of fly eggs and/or larvae available, including no switching, switching, or counter-switching. These findings improve understanding of trophic interactions between mesostigmatic mites and drosophilid flies and highlight the role of dietary experience in shaping predator foraging behaviour.

**Supplementary Information:**

The online version contains supplementary material available at https://doi.org/10.1007/s10493-026-01187-y.

## Introduction

Mesostigmatic mites are free-living species that occupy a variety of niches associated, primarily or secondarily, with soil, litter (e.g., fermentation layers, mosses, bark, dead wood, logs, stumps, tree cavities, and nests) and composters found in a variety of habitats, such as forests, shrublands, dunes, grasslands, agricultural lands, orchards and urban areas (Gulvik 2007; Manu 2008; Salmane and Brumelis 2010; Kaczmarek et al. 2011; Huhta et al. 2012; Manu et al. 2015, 2018; Michalska et al. 2025a; b). Along with other small soil invertebrates, such as springtails, enchytraeids, insects, and oribatid mites, they indirectly contribute to decomposition processes and influence soil quality, including fertility and productivity (Culliney 2013; Adhikari and Hartemink 2016). The majority of them are predators and can be broadly classified as generalist predators of arthropods and nematodes, regulating prey populations through top-down control (Meehan et al. 2021). Some mesostigmatic mites, particularly species inhabiting ephemeral microhabitats, are also phoretic on insects; examples include species such as *Dendrolaelaps quadrisetus* Berlese, *D. disetus* Hirschmann, and members of Macrochelidae and Uropodina, which use insect hosts to disperse to new resources before their current habitats degrade or become depleted (Hunter and Rosario 1988; Liu et al. 2016; Seeman and Walter 2023).

In Diptera, numerous studies have documented associations with mesostigmatic mites, especially among manure-inhabiting species. A number of macrochelid mites are important predators of eggs and younger larval instars of muscid flies (Halliday and Holm 1987; Perotti 2001). Species such as *Macrocheles muscaedomesticae* Scopoli, *M. robustulus* Berlese, and *Glyptholaspis confusa* Foà have been reported as efficient regulators of coprophilous fly populations, with predatory efficiency varying with mite size and developmental stage (Axtell 1961). Macrochelids rely on adult flies for phoretic dispersal between ephemeral habitats and can opportunistically feed on host tissues as ectoparasites (Axtell 1961, 1968; Polak 1996, 1998; Beresford and Sutcliffe 2009; Luong et al. 2017; Durkin et al. 2019). However, compared with the well-documented ectoparasitic associations between adult drosophilid flies and macrochelid mites, especially *M. subbadius* Berlese and *M. muscaedomesticae* (Polak 1996, 1998; Luong et al. 2017; Luong and Subasinghe 2017; Perez-Leanos et al. 2017; Porta et al. 2020; Horn et al. 2023), trophic interactions involving the immature stages of fruit flies and mesostigmatic mites remain poorly studied (Michalska et al. 2025a).

Blattisociid mites are polyphagous predatory mites that inhabit various ecosystems, including stored products, plants, soil, and in association with other arthropods, mites, rodents, birds, and fungi (de Moraes et al. 2015). Among the most studied blattisociid species, *Blattisocius mali* Oudemans is the only genus member with documented associations with drosophilid flies. So far, this species has been recorded on multiple drosophilid hosts under natural conditions (Lehtinen and Aspi 1992; Perez-Leanos et al. 2017; Kerezis et al. 2019). Michalska et al. (2023b) reported an ectoparasitic interaction between *B. mali* and flightless forms of *Drosophila melanogaster* Meigen and *D. hydei* Sturtevant, with females attaching to and feeding on the bodies of adult flies, ultimately leading to higher fly mortality. In another study, Michalska et al. (2023a) confirmed that *B. mali* can prey on eggs and first-instar larvae of these fly species. However, predation by mesostigmatics on later developmental stages of drosophilid fruit flies has not been studied so far.

Generalist predators can consume multiple prey types, which enables them to maintain caloric intake when prey populations fluctuate over time (Whitney et al. 2018). They do not feed randomly among available prey but often show a preference for some prey species over others (Toft 1999; Sigsgaard 2010; Whitney et al. 2018) or for different prey stages. Predatory mites with prior experience of a particular prey species may later attack that prey more rapidly than unfamiliar prey (Christiansen et al. 2016). Inherent or learned prey preferences could be based on prey size (O’Brien et al. 2005; Lai et al. 2011), nutritional value, and ease of capture (Ellis et al. 2012; Mukherjee and Heithaus 2013). These trade-offs may involve costs and benefits (Blackwood et al. 2001). Predators may switch their feeding preferences in response to fluctuations in prey abundance (Murdoch et al. 1975; Visser 1981; Prokopenko et al. 2024). Although prey preference and switching behaviour have been extensively investigated in phytoseiid mites (Khodayari et al. 2016; Alatawi et al. 2019; Fathipour et al. 2020; Dalir et al. 2021; Jahanbazi et al. 2023; Wei et al. 2023), comparable studies on generalist soil-dwelling mesostigmatic predators remain scarce.

For *B. mali*, knowledge is currently limited to interactions with adults and the earliest developmental stages of drosophilids. It remains unclear whether predation patterns differ across later developmental stages, how prior dietary experience shapes subsequent foraging decisions, and whether foraging can be adjusted as prey availability changes.

To address these gaps, this study aimed to determine *B. mali’s* ability to prey on all immature stages of the fruit fly, *D. hydei*, including its prey-stage preferences. Furthermore, we examined for the first time how the predator’s prior diet history shapes its predation rates, prey selection, and prey-switching behaviours. We hypothesised that (i) predation efficiency of *B. mali* differs among developmental stages of *D. hydei*, (ii) prior dietary experience affects prey preference and predation rate, and (iii) *B. mali* exhibits prey-switching behaviour in response to changing prey abundance.

## Materials and methods

### Predator and prey cultures

The wild *D. hydei* Sturtevant (Diptera: Drosophilidae) was used as a prey object, and it was collected from fruit composters at two orchards, in Dąbrowice (51°54’46"N 20°06’54” E) and Nowy Dwór-Pacela (51°52’12"N 20°14’57” E) (Michalska et al. 2025b), located in Łódzkie voivodeship, Poland and was identified using keys from Bächli et al. (2004) and Markow and O’Grady (2006). The colonies of fruit flies were established in 500 ml transparent plastic cups containing a standard fruit fly medium (following the recipe of the BDSC, 2021) that was used in our previous studies (Michalska et al. 2023a; b). The cups were placed in an incubator (Panasonic, Osaka, Japan) set at 25 °C with a 12/12 (L/D) photoperiod. The cup lids were drilled with a 5 cm hole and fitted with non-woven interlining fabric to ensure proper aeration.

Two separate colonies of *Blattisocius mali* (Mesostigmata: Blattisociidae) were also maintained based on the prey type provided: (1) familiar with flies, ‘‘fly-reared predators (FR)”; (2) unfamiliar with flies ‘‘mite-reared predators (MR)”. While *B. mali* was taken from the stock colony that was maintained on the mould mite, *Tyrophagus putrecentiae* (Schrank) in the Department of Plant Protection of Warsaw University of Life Sciences (SGGW) (Michalska et al. 2023a; b) to start these mass rearings. The FR predators’ colony was raised on all stages of *D. hydei*, and the MR predators’ culture was established on mixed stages of *T. putrecentiae*. This acarid mite is regarded as a suitable prey for *B. mali* (Pirayeshfar et al. 2021; Jena et al. 2024, 2026; Michalska et al. 2025c); it is commonly used in mass-rearing of this predator as well as several other generalist predatory mites due to its rapid reproduction, easy maintenance on bran-based substrates, and availability of all developmental stages as prey (Pekas et al. 2017; Pirayeshfar et al. 2021; Asgari et al. 2022; Yazdanpanah and Fathipour 2025; Michalska et al. 2023a, b; c). The rearing of FR predators was carried out in 120 ml plastic containers with a lid having a 4 cm-diameter opening in the middle, secured with non-woven interlining fabric (Fastryga.pl) for aeration. FR predator colonies were reestablished every 15–20 days, after the majority of resources were consumed by the flies in the old mass-rearing. Approximately 100 mites were then collected from the old fly colonies and transferred into new containers containing fresh media and newly established fly colonies that had already begun oviposition. The rearing unit for MR predators consisted of soaked foam platforms (22 cm × 15 cm × 2.5 cm), covered with foil and placed in larger vessels filled with a 1.5 cm layer of water, which provided humidity for mites and prevented them from escaping. Wheat bran was used as a substrate for predatory mites on the platform, and they were fed a mixture of all life stages of *T. putrescentiae* weekly (Michalska et al. 2023a, b; c). Both cultures of FR and MR predators were maintained for a couple of months at 25 °C with a 16L/8D photoperiod in a Panasonic incubator (MIR-154-PE) under 80 ± 5% humidity. *Tyrophagus putrescentiae* used for feeding the MR colony was reared on instant yeast and kept in a desiccator (Chemland, Stargard, Poland) under controlled conditions of 26 °C and 80–85% relative humidity under complete darkness.

### Experimental set-up

The experimental unit consisted of a plexiglass plate measuring 38 mm in length, 30 mm in width, and 4 mm in thickness. A conical hole with an upper diameter of 12 mm and a lower diameter of 5 mm was drilled into the plate (Jena et al. 2026 in press). To create a chamber, a piece of white filter paper was affixed to the bottom of the hole using paraffin wax. A glass coverslip (20 mm by 20 mm) was placed on top of the hole and secured with clips (19 mm size) from both sides to ensure that the mites and the prey could not escape. These clip cages were then placed on a water-soaked foam platform within a large plastic container (35 cm x 30 cm x 10 cm), which was lined with a layer of filter paper. It was given a lid to maintain a high humidity inside. Some holes were also drilled on the lid for aeration.

### Method of obtaining fly eggs

Fly eggs were used as a primary stage to establish cohorts of subsequent developmental stages, first-instar (L1), second-instar (L2), third-instar (L3), and pupae, required for the experiments. To obtain fresh, synchronised fly eggs in bulk, a small Petri dish (5 cm diameter) containing fly media (same as used for mass rearing) and a 1 mm fresh yeast strip in the middle was used to stimulate the flies to lay eggs (Michalska et al. 2023a; b). It was placed inside a 5 L glass jar with a 15 cm bottom diameter and a 10 cm mouth diameter. The jar was covered with non-woven interlining fabric and tightened with a rubber band to allow ventilation while preventing fly escape. Approximately 400–500 flies were released into the jar, and the jar was placed in an incubator at 25 °C under a 12/12 L/D photoperiod. Eggs were collected during the first hour of oviposition, and the age of all other developmental stages, first-, second-, and third-instar larvae and pupae, used in the experiments was also synchronised and did not differ by more than 1 h.

### Predation

In non-choice predation tests, 30 eggs, 20 first- and second-instar larvae, 15 third-instar larvae and 3 pupae per cage per day were given to both types of predators: FR and MR. The number of each immature fly stage offered was decided based on preliminary examinations and size considerations. All immature stages, including pupae, were exposed to predators for 24 h. After each 24-hour exposure period, the consumed and remaining prey items were removed and replaced with freshly collected prey of the same developmental stage. *Blattisocius mali* predation was measured for 11 days. The 11-day period was determined based on pilot tests. It covered almost the entire life span of *B. mali* females when kept in cages. Already on the 11th day, a significant drop in the oviposition was observed, and within the next 3 days of the preliminary tests, all females died. Gravid females of *B. mali* were taken randomly from the colonies, put into clip cages and given a 24-hour starvation period before the start of the experiment. They were then transferred to a new cage and fed appropriate prey at the appropriate stage. This action was repeated every day for 11 days. The number of prey consumed and the number of eggs deposited by the predator were recorded daily. Thirty (*N* = 30) replications, either with (treatment) or without a predator (control), were performed. No visibly damaged or deformed fly eggs were observed in the control cages. No mortality occurred in the fly larval instars in the control, except for the L3. Thus, Abbott’s formula (Abbott 1925) was applied only to L3 because cannibalism was observed in the control group. To estimate predation on fly pupae, each exposed pupa was examined for visible signs of injury, successful adult emergence, and the duration of the pupal stage until emergence of adults.

### Prey preference

For assessing the prey preference of *B. mali*, an equal number of 20 individuals of each fly developmental stage (egg, L1 and L2) was offered in all choices. The preference was evaluated using clip cages in four treatment combinations: (1) 20 eggs + 20 L1, (2) 20 eggs + 20 L2, (3) 20 L1 + 20 L2, and (4) 20 eggs + 20 L1 + 20 L2. Each trial was performed 20 times. In each replication, a single predatory mite was randomly selected from either the FR or the MR culture. It was starved for 24 h, released into each experimental arena, and allowed to feed. The number of each prey type consumed was recorded after 24 h. Manly’s “α” prey preference index was calculated for each combination of predator and prey to measure preference (Manly 1974; Chesson 1983):

1 $$\eqalign{ & = {\text{ }}[\ln (({n_i}_0 - {r_i})/{n_i}_0)]/ \cr & {[_{j = 1}}{\sum ^m}\ln (({n_j}_0 - {r_j})/{n_j}_0)],{\text{ }}i = 1,,,,,,,m,\; \cr}$$

where “α” is Manly’s α index for prey type, “*n*_*i*0_” is the initial number of prey type i, “*r*_*i*_” is the number consumed by the predator for prey type i, “*n*_*j*0_” is the initial number of prey type j, “*r*_*j*_” is the number consumed by the predator for prey type j, while m is the number of prey types in the experiment. The α index takes a value between 0 and 1, and the sum of different prey types is always equal to one. An α value of 0.5 indicates no preference at all, a value greater than 0.5 represents a preference for that particular prey type, while the prey object with a smaller value is least preferred. For each experiment, the α value for each replicate was calculated separately. The mean α value (± 95% Confidence Interval, CI) for each prey type was then determined from these replicate calculations and employed in statistical analyses and data visualisation.

### Prey switching

To estimate prey switching, a single adult female of fly-reared or mite-reared *B. mali* was released into clip cages with the following fly combinations: (1) eggs + L1, (2) eggs + L2 and (3) L1 + L2. Each fly combination was performed at three different ratios of two prey: 20:40, 30:30, and 40:20. Each ratio was replicated 20 times. The predator was removed after 24 h and the numbers of prey types consumed were determined by counting the remaining numbers of each prey type. Murdoch et al. (1969, 1975) defined the null hypothesis of no switching for a system with two prey types as Eq. (1):

2 $${N_1}/{N_2} = c\left( {{H_1}/{H_2}} \right)\;$$

where *H*_*1*_*/H*_*2*_ is the ratio of two prey types in a food offered, *N*_*1*_*/N*_*2*_ is the ratio of two prey types in a food consumed by the predator; under no switching, c is constant and independent of *H*_*1*_*/H*_*2*,_ measuring prey type preference. For calculation purposes (e.g. when N_2_ =0), the data can be transformed to the proportion; then the null hypothesis is written as:

3 $${P_1} = c{F_1}/\left( {1 - {F_1} + c{F_1}} \right)$$

where *P*_*1*_ is the proportion of prey 1 among all prey consumed (= *N*_*1*_*/N*_*1*_ *+ N*_*2*_), and *F*_*1*_ is the proportion of prey 1 in a food offered (= *H*_*1*_*/H*_*1*_ *+ H*_*2*_). Under the condition of switching, c varies with prey availability or encounter and *N*_*1*_*/N*_*2*_ increases faster than linearly with *H*_*1*_*/H*_*2*_ (Murdoch et al.^1975^). Thus, the observed proportion of prey 1 among all prey eaten (*P*_*1*_) is higher than expected when prey 1 is more abundant and lower when prey 1 is rare. However, when the predator disproportionately consumes the less abundant prey type, this pattern is described as counter-switching (or negative switching), indicating a reversal of density-dependent feeding response (Visser 1981; Abrams and Matsuda 1993). For data analysis preference c should be calculated for equally abundant prey 1 and prey 2 (1:1). Then, c will represent the ratio of prey type 1 to prey type 2 consumed, *N*_*1*_*/N*_*2*_; when the value of c = 1, there is no preference; c > 1 indicates a preference for prey type 1, and c < 1 indicates a preference for prey type 2. Following Murdoch et al. (1975), we classified preference as weak when c was close to 1 and less than 3, and as strong when c was greater than 3. For comparative purposes, the magnitude of preference of prey 2 (c < 1) was expressed as 1/c.

To examine switching, we compared the observed and predicted numbers of prey 1 consumed at each of the 20:40 and 40:20 prey 1-to-prey 2 ratios, following the methodology described by Chow and Mackauer (1991) and Mendoza et al. (2024). First, we estimated P1 from Eq. (3) for each replicate in the 30:30 prey 1 to prey 2 ratio. These estimations were then used to calculate the expected P1 in the combination of 40:20 and 20:40. As there was a variation of c among replicates at a 30:30 prey ratio, instead of the arithmetic mean of c, we used the arithmetic mean of P1 for the further calculations of expected predation at any given prey ratio (Chow and Mackauer 1991). In our analysis, fly L2 was assigned as prey 1 in a combination with eggs, while in all other combinations, fly L1 was used as prey 1.

### Cursory observations on the behaviour and spatial distribution of the prey within cages

Since the behaviour and distribution of prey can have a significant impact on the foraging efficiency of a predator, and consequently, on switching (Visser 1981; Higginson and Ruxton 2015), parallel to the experiment on switching, cursorial observations of the behaviour and distribution of fly prey within cages were conducted under similar experimental conditions. Because the distribution of prey could change significantly during the test, observations were made four times, at 2, 6, 12, and 24 h after the placement of prey and the release of mite-reared or fly-reared *B. mali* females into a cage and at the same time intervals in the absence of predators (control). To examine the interactions between the predatory mites and fly larvae during the attack of the mite, several 24-hour starved *B. mali* females were examined. They were put singly in the cages with either first-instar or third-instar larvae. Further methodological details are given in the supplementary material.

### Statistical analysis

Predation rates, oviposition, and survival were analysed using GLMs with a Poisson error distribution and log-link function, as these variables represented non-negative count data. The suitability of the models was evaluated by inspecting residual patterns and checking for overdispersion. Manly’s preference indices (α) were analysed using analysis of variance (ANOVA), with treatment/prey ratio as the fixed factor. Because α is a bounded index, the suitability of ANOVA was verified before inference by checking model assumptions. The normality of residuals was assessed using residual diagnostic plots and the Shapiro–Wilk test, whereas homogeneity of variance was evaluated using Levene’s test. The preference α has been considered significant if the 95% confidence interval for the mean value of α (based on the t-distribution) did not overlap with 0.5 (Chesson 1983). Post hoc comparisons were performed using Tukey’s HSD test. To estimate prey switching, the expected and observed numbers of prey 1 consumed at each 20:40 and 40:20 prey 1-to-prey 2 ratio were analyzed using a Wilcoxon signed-rank test (Chow and Mackauer 1991). Analyses were conducted in R version 4.5.2 using the lmer and emmeans packages (R Core Team 2025).

## Results

### Predation

In non-choice trials conducted for eleven days, gravid females of *B. mali* preyed on all immature stages of *D. hydei* fruit fly, i.e. eggs, first-instar larvae (L1), second-instar larvae (L2) or third-instar larvae (L3) except the pupae of the fly.

**Fig. 1:**
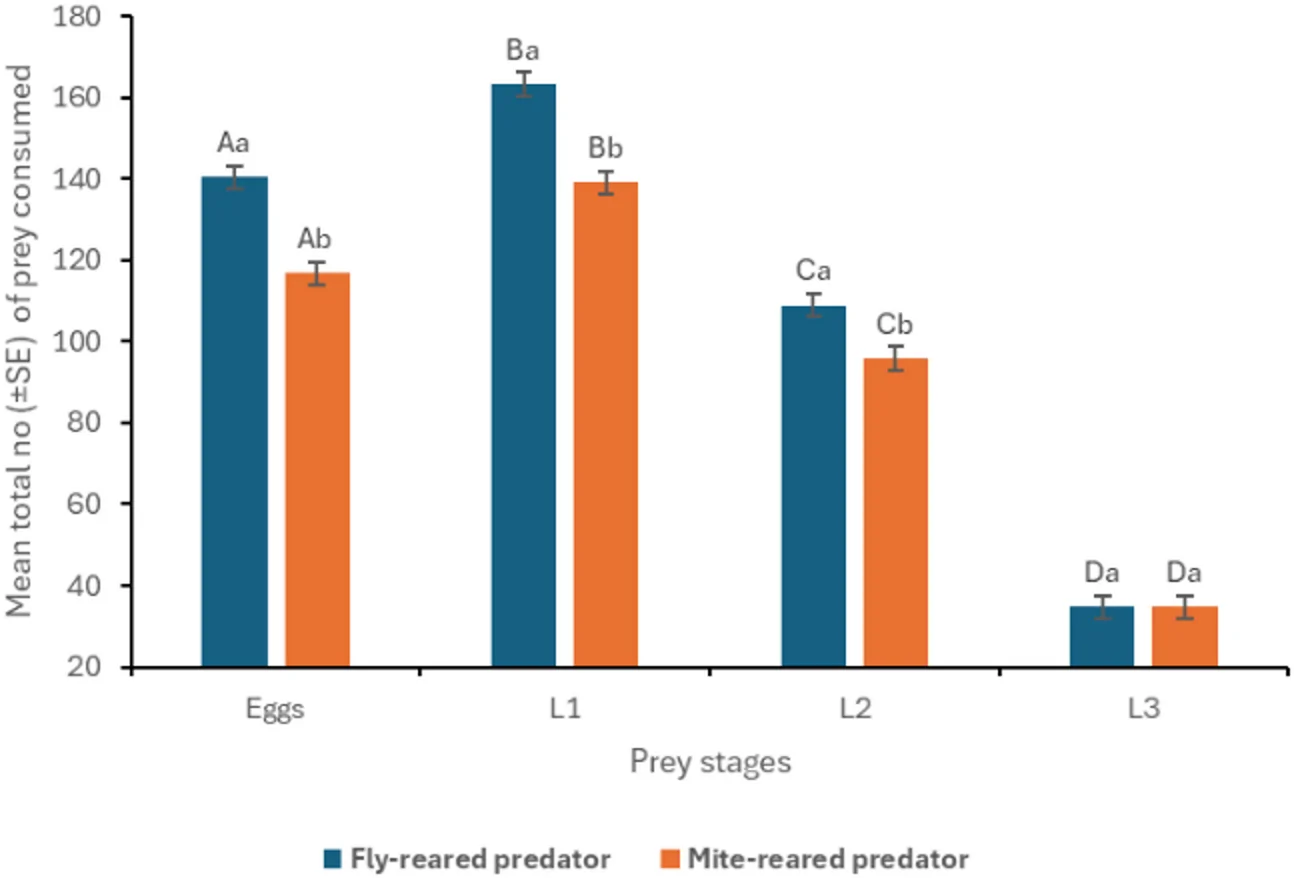
Mean total number (± SE) of *Drosophila hydei* eggs, first-instar larvae (L1), second-instar larvae (L2) and third-instar larvae (L3) consumed by fly- or mite-reared *Blattisocius mali* females during eleven days of observations. Uppercase letters indicate significant differences among prey stages, while lowercase letters indicate significant differences between predator types (fly-reared and mite-reared predators). Bars sharing the same letter are not significantly different, whereas different letters indicate significant differences at *p* < 0.05

The results from GLM analysis showed that the mean total number of prey consumed by a predator during eleven days was significantly influenced by the type of predator (χ^2^ = 21. 3, df = 1, *p* < 0.001), prey stage (χ^2^ = 231.29, df = 4, *p* < 0.001) and predator type x prey stage interaction (χ^2^ = 63.23, df = 4, *p* < 0.001). *Blattisocius mali* females reared on flies or mites consumed in total significantly more fly L1 (*p* < 0.05), followed by eggs (*p* < 0.05), L2 (*p* < 0.05), and then L3 (*p* < 0.05) (Fig. 1). Fly-reared predators ate significantly higher total numbers of fly eggs (*p* < 0.05), L1 (*p* < 0.05) and L2 (*p* < 0.05) than mite-reared females. Both predator types, however, did not differ significantly in the mean total number of L3 consumed over eleven days (*p* ≥ 0.05) (Fig. 1).

None of the pupae exposed to either fly-reared or mite-reared predators for twenty-four hours showed a visible sign of injury. All flies emerged from both treatment and control pupae. Moreover, the mean number of days in the pupal stage until fly eclosion did not differ significantly between control (5.73 ± 0.08 days) and treatment (fly-reared predator: 5.74 ± 0.12 days; mite-reared predator: 5.73 ± 0.12 days) combinations (GLM: χ^2^ = 1.876, df = 2, *p* = 0.8985).

### Oviposition and survival of the predators

**Fig. 2:**
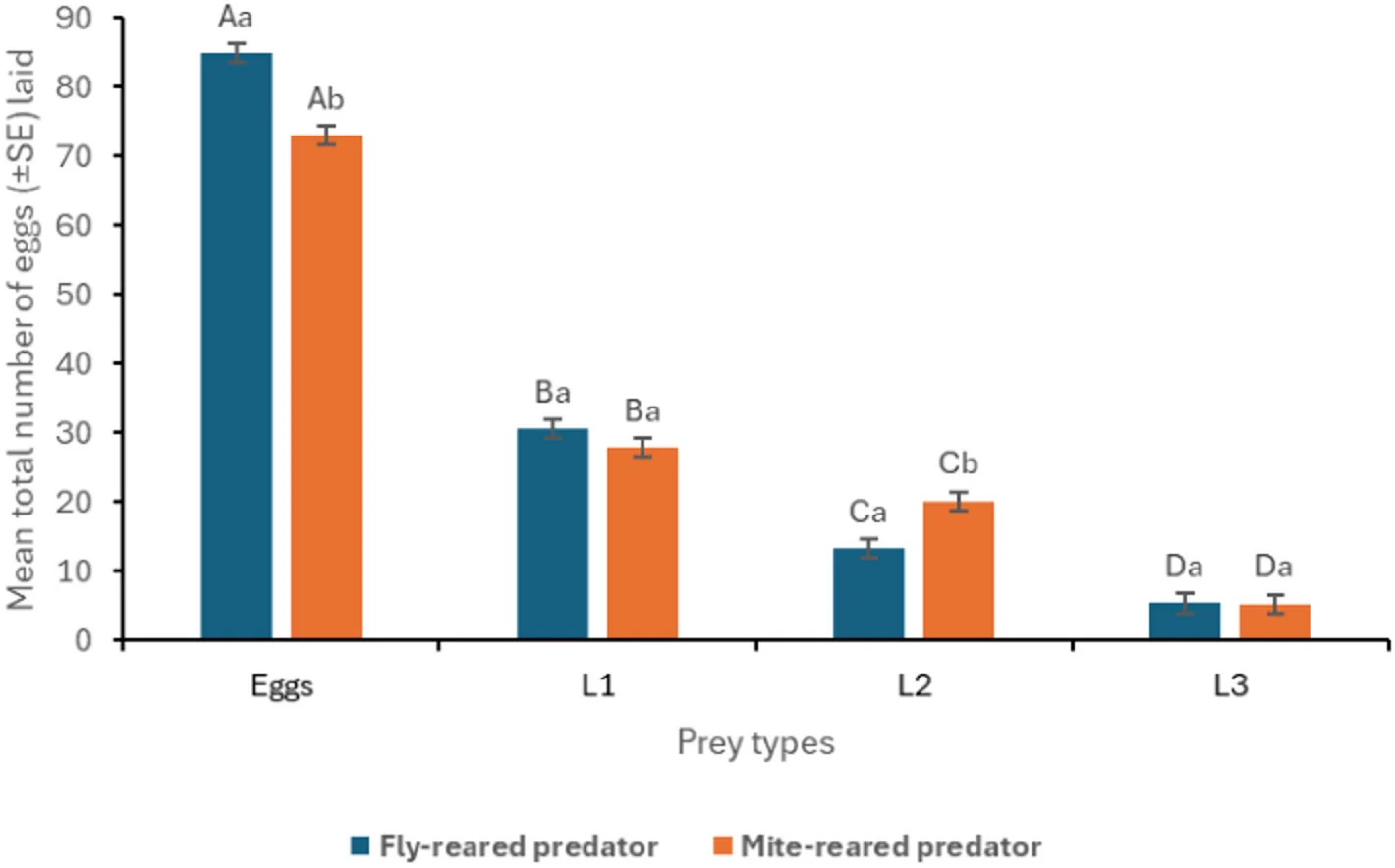
Mean total number of eggs (± SE) laid by *Blattisocius mali* females preying on different immature stages of *Drosophila hydei*, eggs, first-instar larvae (L1), second-instar larvae (L2) and third-instar larvae (L3) during eleven days of observations. Uppercase letters indicate significant differences among prey stages, while lowercase letters indicate significant differences between predator types (fly-reared and mite-reared predators). Bars sharing the same letter are not significantly different, whereas different letters indicate significant differences at *p* < 0.05

In the non-choice trials, the total number of eggs laid by predators during eleven days were significantly influenced by predator type (GLM: χ^2^ = 5.17, df = 1, *p* < 0.036), prey stage (GLM: χ^2^ = 1075.48, df = 4, *p* < 0.001) and predator type x prey stage interaction (GLM: χ² = 12.43, df = 4, *p* < 0.001). Predators deposited much higher mean total number of eggs when they were fed on fly eggs than on L1 (*p* < 0.05), or L2 (*p* < 0.05), and the least number of eggs were laid by predators while preying on L3 (*p* < 0.05) (Fig. 2). When fly eggs were offered as diet, fly-reared predators laid significantly more eggs in total than mite-reared predators (*p* < 0.05) (Fig. 2). On the contrary, fly-reared *B. mali* females deposited significantly fewer eggs than mite-reared *B. mali* when fly L2 was offered as diet (*p* < 0.05). However, the mean total number of eggs deposited by fly-reared and mite-reared predators did not differ significantly when both types of predators were fed on L1 or L3 (*p* ≥ 0.05) (Fig. 2).

**Fig. 3:**
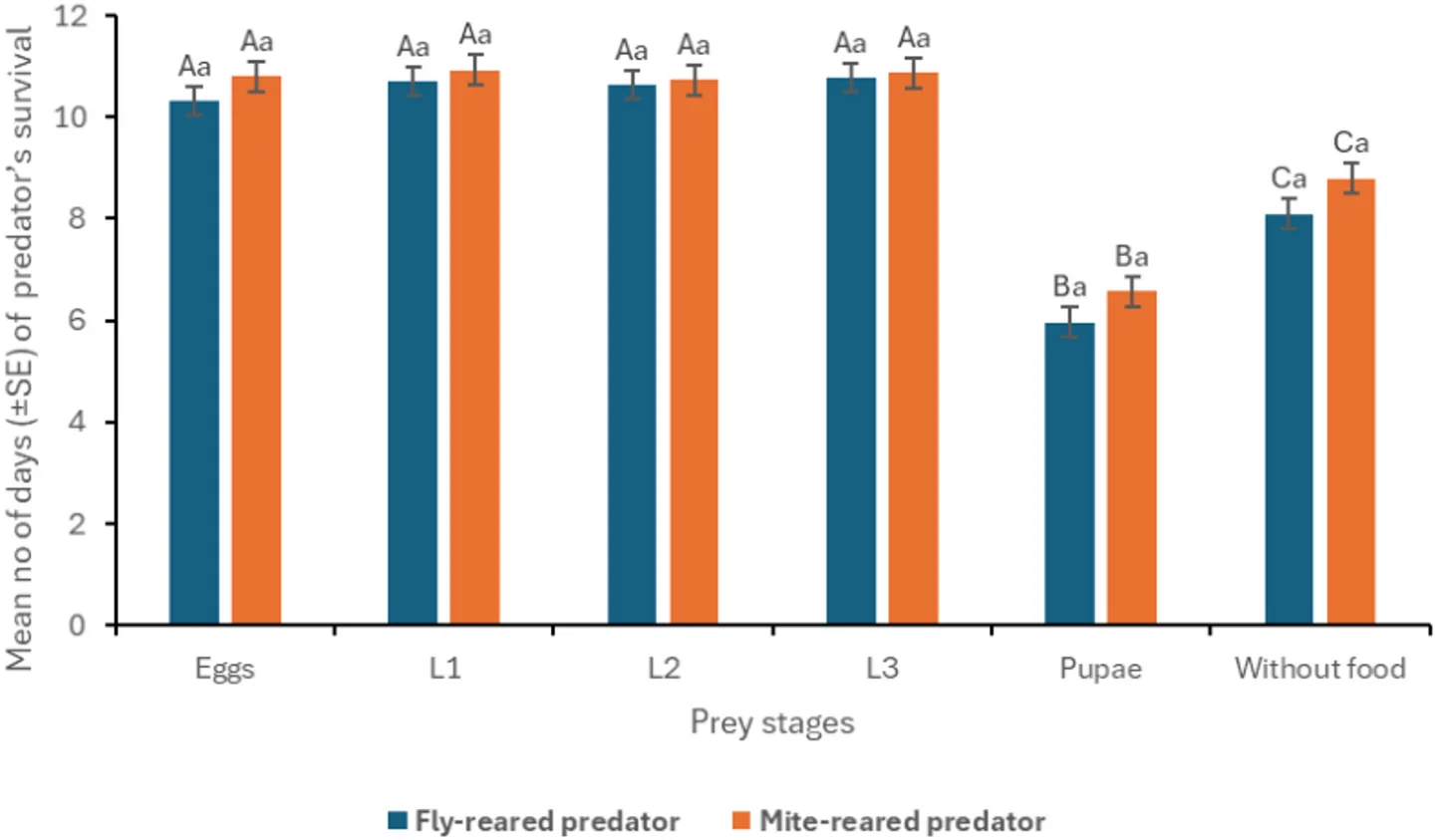
Mean number of days (± SE) that *Blattisocius mali* females survived when kept with *Drosophila hydei* eggs, first-instar larvae (L1), second-instar larvae (L2), third-instar larvae (L3), pupae and without prey during eleven days of observations. Uppercase letters indicate significant differences among prey stages, while lowercase letters indicate significant differences between predator types (fly-reared and mite-reared predators). Bars sharing the same letter are not significantly different, whereas different letters indicate significant differences at *p* < 0.05

The number of days *B. mali* females lived was significantly affected by the predator type (GLM: χ^2^ = 98.1, df = 1, *p* < 0.001), prey (GLM: χ^2^ = 76.26, df = 4, *p* < 0.001) and predator type x prey interaction (GLM: χ² = 192.01, df = 4, *p* < 0.001). In the majority, both fly-reared and mite-reared *B. mali* survived the 11-day-long trial with fly eggs or larvae. There were also no significant differences in the mean number of days the predators lived when exposed to fly eggs, L1, L2 and L3 larvae (*p* ≥ 0.05) (Fig. 3). However, when kept with fly pupae the number of days the predators lived was significantly lower than that of predators being kept with fly eggs or larvae, and was even lower than that of predators kept without prey (*p* < 0.05) (Fig. 3).

### Prey preference

**Fig. 4:**
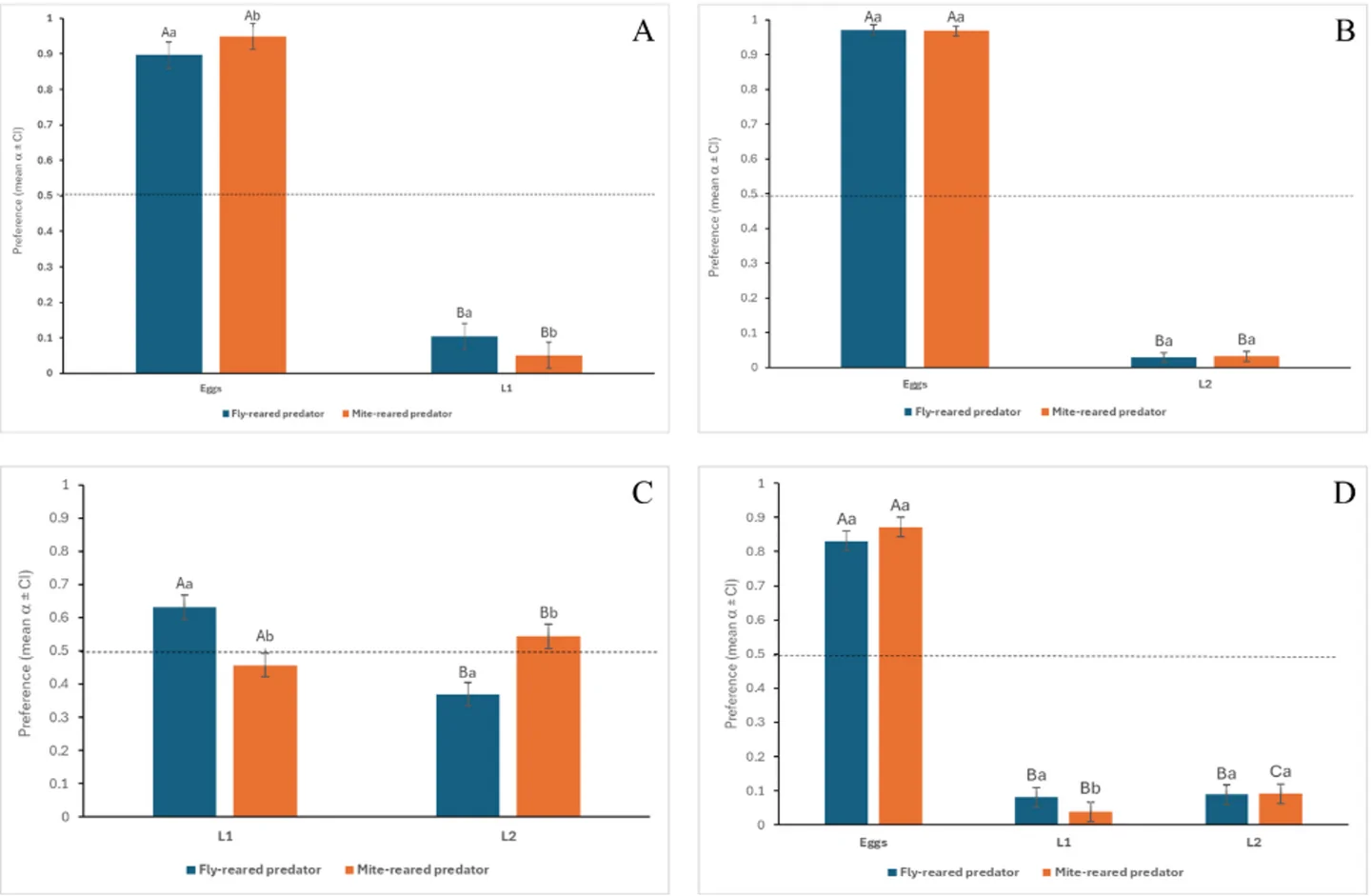
Preference of fly- and mite-reared *Blattisocius mali* females when provided with various prey stage combinations of *Drosophila hydei*: (A) 20 eggs and 20 first-instar larvae (L1); (B) 20 eggs and 20 s-instar larvae (L2); (C) 20 L1 and 20 L2, (D) 20 eggs, 20 L1 and 20 L2. Uppercase and lowercase letters indicate significant differences (*p* < 0.05) among prey stages and predator types, respectively

In the choice experiments, both fly- and mite-reared *B. mali* had a significant preference index in all prey combinations (Fig. 4, α ≠ 0, confidence intervals do not include α = 0.5). The mean prey preference index was significantly affected by the interaction of the prey stage and predator type in the combinations of fly eggs and L1, or fly L1 and L2, while in the combination of fly eggs and L2, only by the prey stage (Table 1). Both predator types exhibited a significantly stronger preference for eggs than larval instars (Fig. 4A, B, D). When provided with eggs and L1, fly-reared *B. mali* females exhibited a significantly weaker preference for eggs and a stronger preference for L1 compared to mite-reared predators (Fig. 4A). However, when exposed to the presence of eggs and L2, both predator types had equally strong preferences for eggs and similarly weak preferences for L2 (Fig. 4B). In the combination with two larval stages of flies, L1 and L2, fly-reared *B. mali* exhibited slightly higher preference for L1 over L2 while mite-reared predator exhibited a weak preference to L2 over L1 (Fig. 4C). Such a preference for L2 over L1 was also exhibited by mite-reared predators in a combination with eggs (Fig. 4D). In this prey composition, both predator types had equally strong preferences for fly eggs. In contrast to mite-reared *B. mali* females, however, fly-reared predators had equally low preferences for L1 and L2 (Fig. 4D).

**Table 1:** The results of two-way ANOVA of the effects of predator type and prey stage on Manly’s preference index, α, for the mite-reared and fly-reared *Blattisocius mali* females when provided with different combinations of prey: eggs, first-instar larva (L1) and second-instar larva (L2) of the fruit fly *Drosophila hydei*

Combination of prey stages	Predator type				Prey stage				Predator type x prey stage			
	F	df	Effect size η²	*p*	F	df	Effect size η²	*p*	F	df	Effect size η²	*p*
eggs vs. L1	0.81	1	0.04	0.8911	2052.59	1	0.99	< 0.0001	7.87	1	0.29	0.0059
eggs vs. L2	0.92	1	0.05	0.7610	17143.94	1	0.99	< 0.0001	0.26	1	0.02	0.6121
L1 vs. L2	2.17	1	0.11	0.9182	23.95	1	0.56	< 0.0001	92.76	1	0.83	< 0.0001
eggs vs. L1 vs. L2	2.11	2	0.09	0.3411	1935.63	2	0.99	< 0.0001	4.06	2	0.29	< 0.0189

### Switching

In the 30:30 prey combination, both types of predators preferred fly eggs to fly larvae. The preference index c of fly-reared *B. mali* calculated for eggs in a combination with L1 and L2 was 5.92 and 5.21, respectively, while in the case of mite-reared predators, 17.21 and 18.76, respectively. By contrast, when both L1 and L2 were offered in equal numbers, fly-reared predators had a weak preference for L1 (c = 1.98) while mite-reared predators showed a weak preference for L2, with c = 1.19.

**Table 2:** Switching of fly-reared and mite-reared *Blattisocius mali* females when given different proportions of *Drosophila hydei* fruit fly eggs, first-instar larvae (L1) or second-instar larvae (L2) in a diet. The observed number of consumed prey types was obtained from the no-switching model

Predator type	No of Prey 1 to Prey 2 offered	Mean (± SE) No. of Prey 1 consumed		Wilcoxon W	*p* value	Switching
		Observed	Expected			
Fly-reared	L1 : Eggs					
	20:40	1.1 ± 0.3	1.8 ± 0.4	247	0.1917	no switching
	30:30:00	2.3 ± 0.6				
	40:20:00	2.8 ± 0.7	5.4 ± 1.2	362	< 0.0001	counter-switching
	L2 : Eggs					
	20:40	0.8 ± 0.4	1.7 ± 0.7	240	0.2519	no switching
	30:30:00	1.8 ± 0.4				
	40:20:00	1.0 ± 0.5	2.6 ± 0.8	232	0.3356	no switching
	L1 : L2					
	20:40	11.0 ± 2.0	17.4 ± 1.4	400	< 0.0001	switching
	30:30:00	4.9 ± 1.5				
	40:20:00	15.6 ± 1.8	20.4 ± 2.1	304	0.0051	counter-switching
Mite-reared	L1 : Eggs					
	20:40	1.3 ± 0.4	0.9 ± 0.2	191	0.8057	no switching
	30:30:00	0.6 ± 0.2				
	40:20:00	1.4 ± 0.6	1.8 ± 0.8	282	0.02668	counter-switching
	L2 : Eggs					
	20:40	0.6 ± 0.2	0.5 ± 0.2	213	0.7159	no switching
	30:30:00	0.9 ± 0.3				
	40:20:00	0.8 ± 0.2	1.1 ± 0.3	238	0.2915	no switching
	L1 : L2					
	20:40	3.6 ± 0.9	11.5 ± 1.1	442	< 0.0001	switching
	30:30:00	2.1 ± 0.4				
	40:20:00	7.9 ± 0.7	12.0 ± 1.5	348	< 0.0001	counter-switching

Table 2 presents the mean numbers of fly eggs, L1 and L2, both observed and predicted by the no-switching model, which were consumed by two types of *B. mali*, when the abundance of the fly stage items offered changed from the proportion 1:1 to the proportion 1:2 or 2:1. As revealed by statistical analysis, *B. mali* exhibited switching only when both fly L1 and L2 were offered as prey (Table 2). In a combination of 20 L1:40 L2, fly-reared and mite-reared predators switched to L2, consuming a significantly lower number of L1 than expected under the no-switch model. However, when L1 were more abundant (a combination of 40 L1:20 L2), fly-reared and mite-reared *B. mali* consumed significantly fewer L1 than expected by the model, indicating counter-switching to L2 (Table 2). Also, when L1 was more abundant in combination with eggs (40 L1:20 eggs), both predator types (instead of consuming disproportionately more L1 than predicted by the no-switching model) ate significantly fewer L1, indicating counter-switching to eggs. In the remaining combinations, the observed and predicted mean number of prey consumed by both fly-reared and mite-reared *B. mali* did not differ significantly, which indicated no prey switching (Table 2).

**Table 3:** Mean number (± SE) of prey consumed by fly-reared and mite-reared *Blattisocius mali* females at different ratios of eggs and first-instar larvae (L1), eggs and second-instar larvae (L2) and L1 and L2. Different letters indicate significant differences (*P* < 0.05) between both predator types

	Fly-reared predators	Mite-reared predators	χ″	df	*p*-value
Eggs : L1					
Eggs to L1 ratio	Mean no. ± SE of eggs consumed	Mean no. ± SE of eggs consumed			
20:40	15.00 ± 0.40 a	12.20 ± 0.21 b	10.21	38	< 0.001
30:30:00	13.45 ± 0.38 a	12.80 ± 0.24 a	2.31	38	0.291
40:20:00	14.85 ± 0.33 a	12.70 ± 0.38 b	12.14	38	< 0.001
L1 to eggs ratio	Mean no. ± SE of L1 consumed	Mean no. ± SE of L1 consumed			
20:40	2.75 ± 0.35 a	1.35 ± 0.21 b	6.23	38	0.011
30:30:00	2.15 ± 0.51 a	0.25 ± 0.10 b	7.51	38	< 0.001
40:20:00	1.10 ± 0.27 a	1.30 ± 0.18 a	1.54	38	0.542
Eggs : L2					
Eggs to L2 ratio	Mean no. ± SE of eggs consumed	Mean no. ± SE of eggs consumed			
20:40	13.40 ± 0.35 a	12.20 ± 0.32 b	43.12	38	< 0.001
30:30:00	14.15 ± 0.37 a	11.90 ± 0.28 b	27.11	38	< 0.001
40:20:00	16.40 ± 0.40 a	11.80 ± 0.63 b	15.98	38	< 0.001
L2 to eggs ratio	Mean no. ± SE of L2 consumed	Mean no. ± SE of L2 consumed			
20:40	1.00 ± 0.32 a	0.75 ± 0.19 a	1.82	38	0.328
30:30:00	2.60 ± 0.41 a	0.65 ± 0.21 b	10.63	38	< 0.001
40:20:00	0.75 ± 0.23 a	0.55 ± 0.14 a	0.91	38	0.766
L1 : L2					
L1 to L2 ratio	Mean no. ±SE of L1 consumed	Mean no. ±SE of L1 consumed			
20:40	11.00 ± 0.83 a	3.55 ± 0.51 b	32.11	38	< 0.001
30:30:00	13.55 ± 1.10 a	4.40 ± 0.48 b	26.08	38	< 0.001
40:20:00	15.55 ± 1.00 a	7.85 ± 0.55 b	28.38	38	< 0.001
L2 to L1 ratio	Mean no. ±SE of L2 consumed	Mean no. ±SE of L2 consumed			
20:40	13.05 ± 1.02 a	7.50 ± 0.43 b	20.37	38	< 0.001
30:30:00	7.90 ± 1.05 a	5.45 ± 0.37 b	7.19	38	0.005
40:20:00	5.35 ± 0.41 a	4.60 ± 0.51 b	4.11	38	0.043

As shown in Table 3, both types of *B. mali* differed in the number of fly eggs and larvae consumed in all prey ratios offered. In a combination with fly eggs and L1, fly-reared predators consumed significantly more prey than mite-reared ones, except for the prey ratio of 30 eggs:30 L1, and 40 L1:20 eggs, when there were no significant differences between both types of predators. Similarly, in other prey ratio combinations, fly-reared predators consumed more prey, except for the prey ratios when L2 were more abundant than eggs (40 L2:20 eggs), or L2 were more abundant than L1 (40 L2:20 L1), in which case there were no significant differences in the number of eaten L2 between the two predator types.

As revealed by the cursory observations of prey within a cage, the fly larvae tended to form aggregations. A detailed description of their distribution and behaviour is provided in the supplementary material. In contrast to second-instar larvae, first-instar larvae often formed tight tangles of entwined individuals that performed continuous, multidirectional movements (Video S1). The clusters of second-instar larvae were less compact, with individuals adhering to each other longitudinally and moving forward in the same or opposite direction. In a combination with eggs, first-instar larvae were found both on the cage bottom with eggs and throughout the remaining parts of the cage (Table S1). By comparison, second-instar larvae, 2 h after release to the bottom of a cage, all migrated to the cell walls and the underside of the cover slip (Table S2). In a combination of both L1 and L2, larval aggregations were found all over the cage (Table S3). When first-, second-, and third-instar (L3) larvae moved alone outside aggregations, they often defended themselves against *B. mali* females by attempting to rush away and engaging in vigorous wrestling with the attacking predator (Videos S3, S4).

## Discussion

In this study, *B. mali* females preyed on eggs and all three larval instars of the wild strain of *D. hydei*. The predator’s previous diet history, mass-rearing on fruit flies or acarid mites, significantly affected both predation rate and its preference for a particular immature fly stage. Moreover, depending on the proportion of fly eggs and/or larvae in a diet, *B. mali* females behaved differently, exhibiting either no-switching, switching or counter-switching to the more abundant prey stage. It is one of the few detailed studies of a predatory mite feeding on all immature stages of fruit flies. Previous studies referred to predation by *Pergamasus longicornis* Berlese on the final larval stage *of D. melanogaster* (Bowman 1987), and *B. mali* on eggs of the flightless strain of *D. melanogaster* and *D. hydei* (Michalska et al. 2023a) and the first- instar larvae of flightless *D. hydei* (Michalska et al. 2023b). All other reports referred to macrochelid mites’ predation on immature muscid flies and on adults of muscid and drosophilid flies (Ito 1977; Halliday and Holm 1987; Perotti 2001).

In our study, *B. mali* most likely did not feed on *D. hydei* pupae, as all flies exposed to the mite had emerged and did not delay emergence compared with the control. Moreover, the mortality of the predator kept with the pupae was much higher than with other *D. hydei* immature stages, and even higher than that of starved predators. The latter finding could have been linked to the young age of the pupa tested and the increased activity of the predatory mite exposed to it. Our unpublished observations revealed that, unlike older *D. hydei* pupae, young ones were frequently examined by predators that attempted to insert their chelicerae into their bodies. It is likely that the young pupae lack a fully developed pupal cuticle and/or produce chemical cues that stimulate mite investigation. However, cuticle thickness may prevent predators from successfully feeding, leading to increased energetic costs of pupa attendance and penetration attempts. Although we did not observe any visible signs of injury on the pupa’s body after 1 day of exposure to a predatory mite, further studies on the viability of flies after emergence are undoubtedly needed.

As revealed by our study, the previous diet has a marked impact on the predation rate of *B. mali*. In eleven-day-long non-choice tests, which covered almost the entire lifespan of caged *B. mali* females, predators that were previously reared on flies consumed significantly more fly eggs or larvae than mite-reared predators, except for the third-instar larva, on which the predation rates were similar between the two groups. This pattern suggests that exploiting a novel prey type may require physiological and behavioural adjustments, resulting in lower predation efficiency than when predators are exposed to that prey over multiple generations. The influence of rearing history and early-life experience on predator performance has been reported for several predatory mite species (Castagnoli and Simoni 1999; Schausberger et al. 2020, 2021). For example, populations of *Neoseiulus persimilis* Athias-Henriot with different nutritional histories exhibited distinct functional and numerical responses when feeding on tetranychid mites (Castagnoli and Simoni 1999). Likewise, prior exposure to thrips improved prey recognition and enhanced the performance of *Amblyseius swirskii* Athias-Henriot, leading to faster population growth and stronger suppression of thrips populations (Schausberger et al. 2020). However, prey experience does not always improve predator performance. Jensen et al. (2019) found that exposure to the risky prey *Protaphorura fimata* Gisin induced prey aversion in *Gaeolaelaps aculeifer* Canestrini, reducing both prey preference and predation efficiency. It is also likely that, when mass-reared on flies, *B. mali* rarely preyed on, or did not prey upon third-instar fly larvae and therefore, in our non-choice test, consumed a similar number of these larvae as predators previously reared on acarid mites.

The non-choice test also revealed that both types of predators consumed more first-instar fly larvae than eggs, fewer second-instar larvae than eggs, and the fewest number of third-instar larvae. Generally, the larger the prey, the greater the potential food portion a predator may obtain from it, though it may also pose greater attack difficulties and greater challenges in subduing and feeding. For example, Halliday and Holm (1987) demonstrated that macrochelid predatory mites differed markedly in their ability to attack bushfly larvae depending on prey developmental stage, with early stages being far more vulnerable than later instars. Larger fly larvae were more difficult to subdue and suffered lower mortality, despite their higher potential nutritional value.

Preliminary observations revealed that *D. hydei* larvae can vigorously defend themselves against attacks by *B. mali* females. The larvae often forced the predator to pursue them, and when the predator succeeded in jumping onto them, they twisted and bent their bodies in various directions to dislodge it. Such behaviour likely increased the energetic costs to the predator when attacking fly larvae compared with attacking fly eggs. Interestingly, our unpublished data indicate that first-instar larvae of *D. hydei* have a lower biomass than fly eggs. Consequently, to meet their nutritional requirements, *B. mali* females would be expected to consume a greater number of first-instar larvae than eggs, as observed in our experiments. Despite this, females feeding on first-instar larvae laid nearly half as many eggs as those feeding on fly eggs, and their oviposition rate declined even further when they fed on second- or third-instar larvae. The significantly higher egg production observed in *B. mali* females fed on *D. hydei* eggs may indirectly indicate that fly eggs possess a higher nutritional value than fly larvae. Alternatively, this difference may reflect lower handling costs for eggs than for larvae. These factors, which are not mutually exclusive, could explain the strong preference for eggs over first- and second-instar larvae observed in both two-choice and three-choice tests. It should also be noted that females may reduce or cease egg production when the available prey is unsuitable for their offspring (Fréchette et al. 2006; Ferrer et al. 2011). Therefore, further studies are needed to determine whether the immature stages of *B. mali*, similarly to adult females, are capable of feeding on the eggs or larvae of *D. hydei*.

Interestingly, when given a choice between the two, the fly egg and fly first- or second-instar larvae, both predator types showed opposite preferences: mite-reared predators preferred second-instar larvae, while fly-reared predators preferred first-instar larvae. Thus, counterintuitively, naïve, mite-reared predators chose a bigger sized prey type than their experienced counterparts. Our preliminary observations on the distribution of fly larvae and eggs in a cage shed more light on this issue. Although walking first-instar larvae generally moved more slowly than second-instar larvae, in cage-aggregated groups, both types of larvae behaved differently. While first-instar larvae tended to form a tight tangle of highly active, entwining individuals, clusters of second-instar larvae were less compact, with individuals adhering to each other longitudinally and moving forward in the same or opposite direction. Consequently, grouped second-instar larvae may have been easier to attack and more readily selected by less experienced mite-reared predators. However, preying on this fly stage is costly, as shown by the non-choice test. Fly-reared predators consumed more second-instar larvae than mite-reared predators but had lower egg production (although on fly eggs and first-instar diet, they outperformed mite-reared predators in consuming these prey types and producing eggs). It is likely that, even if the second-instar larvae are easier to attack, they may require greater expenditure of energy to be subdued. Thus, when given a choice, *B. mali* individuals that had already experienced hunting second-instar larvae in mass-rearing conditions may have preferred first-instar to second-instar larvae. However, to fully explain this phenomenon, further comparative observations of *B. mali*’s predatory behaviour towards first- and second-instar fly larvae, including handling time, attack latency and success rate, are necessary.

In our further experiment on prey switching, in which equal prey numbers were increased to 30:30, *B. mali* again showed a very high preference to fly eggs in combination with either first- or second-instar larvae, and a weak preference in combination with first- and second-instar larvae. Moreover, mite-reared predators exhibited a much stronger preference for fly eggs than fly-reared predators. It is likely that the mite-reared *B. mali* females consumed mostly fly eggs because those required less effort and hunting skill than fly larvae, allowing the naïve predatory females to quickly satisfy their hunger and convert the energy into egg production. This finding indicates that diet history not only influences whether and how many novel prey a predator consumes (Lv et al. 2016; Schuldiner-Harpaz et al. 2016), but also which stage of that prey it prefers.

Only when offered two larval instars did both fly- and mite-reared *B. mali* females exhibit prey switching, consuming disproportionately fewer first-instar larvae in favour of second-instar larvae when the former became rare (20 L1:40 L2). However, because predators were tested only at two biased prey-stage ratios (1:2 and 2:1), switching in other prey combinations at more extreme ratios cannot be ruled out (Murdoch 1969). Prey switching is generally attributed to cognitive and behavioural constraints in predator foraging, including search-image formation and learning, which can increase detection and capture efficiency for abundant prey types (Allen and Greenwood 1988; Endler 1991; Endler and Rojas 2009; Alexander et al. 2022; Prokopenko et al. 2024). Also, in our experiment, where there were twice as many second-instar larvae as first-instar larvae, the switching in both types of *B. mali* may therefore have resulted from improved exploitation of the more abundant prey stage. Similarly, one would expect a shift toward second-instar larvae in a trial where fly eggs were rare. However, our observations indicated that already 2 h after placing the larvae and eggs on the bottom of the cage, virtually all second-instar larvae had migrated to the walls and the underside of the coverslip. This led to spatial separation of the two prey types. Given that eggs are the preferred prey type, the predator most likely stayed primarily on the bottom of the cage, where second-instar larvae were absent, and presumably preyed on the larvae infrequently enough to perfect the hunt and switch. Nevertheless, switching to second-instar larvae at more extreme prey ratios cannot be ruled out. It must also be stressed that, in our experiment, fly eggs were relatively easy prey, presumably not requiring much learning to detect and consume them. Therefore, as expected, when eggs were more abundant than other prey types, switching to eggs did not occur. In contrast, when first-instar larvae were more abundant, predators exhibited counter-switching. This may be related to the previously mentioned high mobility of the aggregated first-instar larvae, which, especially at the higher densities, may have discouraged *B. mali* from hunting them in favour of rarer eggs or second-instar larvae.

In conclusion, our study demonstrated a high predatory potential of *B. mali* against different developmental stages of fruit flies. Although the mite showed a preference for fly eggs, it also fed and reproduced on all three larval instars. Therefore, *B. mali* may play an important role not only in regulating fruit fly populations but also in the biological control of invasive pest species such as *D. suzukii* Matsumura (Deconninck et al. 2024). Interestingly, *B. mali* performed better when previously reared on fruit flies rather than on acarid mites. This finding should be considered when developing mass-rearing protocols for this predator intended for biological control applications. Nevertheless, further research is required to evaluate the practical implications of this effect under large-scale production conditions.

It should be emphasised that under natural conditions, fruit fly eggs and larvae are often less accessible to predators than under laboratory conditions. On decaying plant material, eggs may be buried within the substrate, while larvae can penetrate deeper layers or even become completely submerged, depending on substrate consistency, larval instar, fly species, light conditions, and surface disturbance (Carton and David 1985; Albornoz and Domínguez 1987; Godoy-Herrera 1994; Kim et al. 2017; Dombrovski et al. 2020). Similarly, in our laboratory cultures, eggs and larvae of *D. hydei* were frequently found buried in the culture medium, whereas individuals present on the surface were relatively sparse and scattered.

Preliminary observations indicate that *B. mali* can locate and feed on buried *D. melanogaster* eggs (Michalska et al. 2023c). However, its efficiency in locating and attacking fruit fly larvae concealed within the substrate remains unknown. Larval burrowing behaviour is considered an important fruit fly adaptation that facilitates more efficient exploitation of food resources, reduces competition, prevents desiccation, and provides protection from natural enemies, including parasitoids (Godoy-Herrera 1994; Kim et al. 2017). Therefore, future studies should investigate the predatory performance of *B. mali* under more complex, naturalistic conditions, including substrates (e.g. decaying plant material or fruit fly culture medium) that support egg burial and larval burrowing. Such studies would complement the present findings and contribute to a better understanding of the ecological role and biological control potential of this predator.

## Supplementary Information

Below is the link to the electronic supplementary material.

Supplementary Material 1

Supplementary Material 2

Supplementary Material 3

Supplementary Material 4

Supplementary Material 5

## Data Availability

The datasets generated during and/or analysed during the current study are available from the corresponding author on reasonable request.

## References

[CR1] Abbott WS (1925) A method of computing the effectiveness of an insecticide. J Econ Entomol 18:265–267. 10.1093/jee/18.2.265a

[CR2] Abrams P, Matsuda H (1993) Effects of adaptive predatory and anti-predator behaviour in a two-prey—one-predator system. Evol Ecol 7(3):312–326. 10.1007/BF01237749

[CR3] Adhikari K, Hartemink AE (2016) Linking soils to ecosystem services—A global review. Geo 262:101–111. 10.1016/j.geoderma.2015.08.009

[CR4] Alatawi FJ, Mushtaq HMS, Mirza JH, Kamran M (2019) Predation efficiency and preference of lab-reared and field-collected populations of predatory mite *Cydnoseius negevi* (Acari: Phytoseiidae) on two mite pest species *Oligonychus afrasiaticus* and *Tetranychus urticae* (Acari: Tetranychidae). Int J Pest Manag 65(4):363–369. 10.1080/09670874.2018.1529340

[CR5] Albornoz J, Domínguez A, Alcorta E, Santiago E (1987) Chromosomal effects in egg laying of *Drosophila melanogaster* under different conditions. Her 58(3):457–462. 10.1038/hdy.1987.75

[CR6] Alexander ME, Skein L, Robinson TB (2022) Rapid learning in a native predator shifts diet preferences towards invasive prey. Biol Lett 18(3):20210655. 10.1098/rsbl.2021.0655 PMC8905153 35259942

[CR7] Allen JA, Greenwood JJD (1988) Frequency-dependent selection by predators. Philosophical Trans Royal Soc Lond Biol Sci 319(1196):485–503 10.1098/rstb.1988.0061 2905488

[CR8] Asgari F, Safavi SA, Moayeri HRS (2022) Life table parameters of the predatory mite, *Blattisocius mali* Oudemans (Mesostigmata: Blattisociidae), fed on eggs and larvae of the stored product mite, *Tyrophagus putrescentiae* (Schrank). Egypt J Biol Pest Control 32(1):118. 10.1186/s41938-022-00616-5

[CR9] Axtell RC (1961) New records of North American Macrochelidae (Acarina: Mesostigmata) and their predation rates on the house fly. Ann Entomol Soc Am 54(5):748–748. 10.1093/aesa/54.5.748

[CR10] Axtell RC (1968) Integrated house fly control: populations of fly larvae and predaceous mites, *Macrocheles muscaedomesticae*, in poultry manure after larvicide treatment. J Econ Entomol 61:245–249. 10.1093/jee/61.1.245 5636841

[CR11] Bächli G, Vilela CR, Andersson S, Saura A (2004) The Drosophilidae (Diptera) of Fennoscandia and Denmark. Fauna Entomologica Scandinavica 39. Leiden: Brill, vol 39. Brill, Leiden, New York

[CR12] BDSC (2021) Bloomington *Drosophila* Stock Center (BDSC) Cornmeal Food. Department of Biology, Indiana University. https://bdsc.indiana.edu/information/recipes/bloomfood.html. Accessed 6 June 2026

[CR13] Beresford DV, Sutcliffe JF (2009) The effect of Macrocheles muscaedomesticae and M. subbadius (Acarina: Macrochelidae) phoresy on the dispersal of Stomoxys calcitrans (Diptera: Muscidae). Syst Appl Acarol 23:1–30. 10.11158/saasp.23.1.1

[CR14] Blackwood JS, Schausberger P, Croft BA (2001) Prey-stage preference in generalist and specialist phytoseiid mites (Acari: Phytoseiidae) when offered *Tetranychus urticae* (Acari: Tetranychidae) eggs and larvae. Env Ento 30(6):1103–1111. 10.1603/0046-225X-30.6.1103

[CR15] Bowman CE (1987) Studies on feeding in the soil predatory mite *Pergamasus longicornis* (Berlese)(Mesostigmata: Parasitidae) using dipteran and microarthropod prey. Exp Appl Acarol 3(3):201–206

[CR16] Carton Y, David JR (1985) Relation between the genetic variability of digging behavior of *Drosophila* larvae and their susceptibility to a parasitic wasp. Behav Genet 15(2):143–154 10.1007/BF01065895 3838072

[CR17] Castagnoli M, Simoni S (1999) Effect of long-term feeding history on functional and numerical response of *Neoseiulus californicus* (Acari: Phytoseiidae). Exp Appl Acarol 23(3):217–234. 10.1023/A:1006066930638

[CR18] Chesson J (1983) The estimation and analysis of preference and its relatioship to foraging models. Ecol 64(5):1297–1304. 10.2307/1937838

[CR19] Chow A, Mackauer M (1991) Patterns of host selection by four species of Aphidiid (Hymenoptera) parasitoids: influence of host switching. Ecol Entomol 16:403–410. 10.1111/j.1365-2311.1991.tb00233.x

[CR20] Christiansen IC, Szin S, Schausberger P (2016) Benefit-cost trade-offs of early learning in foraging predatory mites *Amblyseius swirskii*. Sci Rep 6(1):23571. 10.1038/srep23571 PMC4804281 27006149

[CR21] R Core Team (2025) R: A language and environment for statistical computing. R Foundation for Statistical Computing. https://www.r-project.org/. Accessed 20 March 2026

[CR22] Culliney TW (2013) Role of arthropods in maintaining soil fertility. Agri 3:629–659. 10.3390/agriculture3040629

[CR23] Dalir S, Hajiqanbar H, Fathipour Y, Khanamani M (2021) A comprehensive picture of foraging strategies of *Neoseiulus cucumeris* and *Amblyseius swirskii* on western flower thrips. Pest Manag Sci 77(12):5418–5429 10.1002/ps.6581 34329533

[CR24] de Moraes GJ, Venancio R, dos Santos VLV, Paschoal AD (2015) Potential of Ascidae, Blattisociidae and Melicharidae (Acari: Mesostigmata) as Biological Control Agents of Pest Organisms. Prospects for Biological Control of Plant Feeding Mites and Other Harmful Organisms. Springer International Publishing, Cham, Switzerland

[CR25] Deconninck G, Boulembert M, Eslin P, Couty A, Dubois F, Gallet-Moron E, Pincebourde S, Chabrerie O (2024) Fallen fruit: a backup resource during winter shaping fruit fly communities. Agric For Entomol 26(2):232–248. 10.1111/afe.12610

[CR26] Dombrovski M, Kuhar R, Mitchell A, Shelton H, Condron B (2020) Cooperative foraging during larval stage affects fitness in *Drosophila*. J Comp Physiol A 206(5):743–755. 10.1007/s00359-020-01434-6 PMC7392940 32623493

[CR27] Durkin ES, Proctor H, Luong LT (2019) Life history of *Macrocheles muscaedomesticae* (Parasitiformes: Macrochelidae): new insights on life history and evidence of facultative parasitism on *Drosophila*. Exp Appl Acarol 79(3):309–321 10.1007/s10493-019-00431-y 31673886

[CR28] Ellis JC, Allen KE, Rome MS, Shulman MJ (2012) Choosing among mobile prey species: Why do gulls prefer a rare subtidal crab over a highly abundant intertidal one? J Exp Mar Biol Ecol 416:84–91. 10.1016/j.jembe.2012.02.014

[CR29] Endler JA (1991) Variation in the appearance of guppy color patterns to guppies and their predators under different visual conditions. Vis Res 31(3):587–608 10.1016/0042-6989(91)90109-i 1843763

[CR30] Endler JA, Rojas B (2009) The spatial pattern of natural selection when selection depends on experience. Am Nat 173(3):E62–E78 10.1086/596528 19199522

[CR31] Fathipour Y, Maleknia B, Bagheri A, Soufbaf M, Reddy GV (2020) Functional and numerical responses, mutual interference, and resource switching of *Amblyseius swirskii* on two-spotted spider mite. Bio Cont 146:104266. 10.1016/j.biocontrol.2020.104266

[CR32] Ferrer A, Corbani AC, Dixon AF, Hemptinne JL (2011) Egg dumping by predatory insects. Physiol Entomol 36(3):290–293. 10.1111/j.1365-3032.2011.00780.x

[CR33] Fréchette B, Coderre D, Lucas É (2006) *Chrysoperla rufilabris* (Neuroptera: Chrysopidae) females do not avoid ovipositing in the presence of conspecific eggs. Biol Control 37(3):354–358. 10.1016/j.biocontrol.2005.12.012

[CR34] Godoy-Herrera R, Santander R, Figueroa J (1994) A developmental and biometrical analysis of larval photoresponse of Drosophila. Anim Behav 48(2):251–262. 10.1006/anbe.1994.1239

[CR35] Gulvik ME (2007) Mites (Acari) as indicators of soil biodiversity and land use monitoring: A review. Pol J Ecol 55:415–450

[CR36] Halliday RB, Holm E (1987) Mites of the family Macrochelidae as predators of two species of dung-breeding pest flies. Entom 32(4):333–338

[CR37] Higginson AD, Ruxton GD (2015) Foraging mode switching: the importance of prey distribution and foraging currency. Anim Behav 105:121–137. 10.1016/j.anbehav.2015.04.014

[CR38] Horn CJ, Liang C, Luong LT (2023) Parasite preferences for large host body size can drive overdispersion in a fly-mite association. Int J Parasitol 53(7):327–332. 10.1016/j.ijpara.2023.03.003 37054865

[CR39] Huhta V, Pietikäinen AS, Penttinen R (2012) Importance of dead wood for soil mite (Acarina) communities in boreal old-growth forests. Soil Org 84(3):499–512. https://doi.org/10.25674

[CR40] Hunter PE, Rosario RMT (1988) Associations of Mesostigmata with other arthropods. Ann Rev Entomol 33:393–417. 10.1146/annurev.en.33.010188.002141

[CR41] Ito Y (1977) Predatory activity of mesostigmatid mites (Acarina: Mesostigmata) for house fly eggs and larvae under feeding of nematodes. Japan Soc Med Entomol Zool 28:167–173

[CR42] Jahanbazi M, Sedaratian-Jahromi A, Ghane-Jahromi M (2023) Comparative study of predation, preference and switching behaviors of two predatory mite *Neoseiulus californicus* and *Amblyseius swirskii* (Acari: Phytoseiidae). Int J Pest Manag 69(4):366–376

[CR43] Jena MK, Michalska K, Studnicki M (2024) The impact of humidity on the functional response of Blattisocius mali (Acari: Blattisociidae) preying on the acarid mite Tyrophagus putrescentiae. Sci Rep 14(1):28051. 10.1038/s41598-024-78997-w PMC11564752 39543199

[CR93] Jena MK, Michalska K, Studnicki M (2026 in press). Effect of temperature on life table parameters of the predatory soil mite Blattisocius mali (Acari: Blattisociidae) fed on Tyrophagus putrescentiae (Acari: Acaridae). Sci Rep. 10.1038/s41598-026-66347-x PMC13550589 42706385

[CR44] Jena MK, Studnicki M, Sochacki D, Michalska K (2026) Evaluation of the predatory soil mite *Blattisocius mali* (Acari: Blattisociidae) as a biocontrol agent of the acarid mite *Tyrophagus putrescentiae* (Acari: Acaridae) infesting stored flower bulbs. Bio Cont 71:559–571. 10.1007/s10526-026-10395-9

[CR45] Jensen K, Toft S, Sørensen JG, Sigsgaard L, Kristensen TN, Overgaard J, Holmstrup M (2019) Prey-specific experience affects prey preference and time to kill in the soil predatory mite *Gaeolaelaps aculeifer* Canestrini. Biol Cont 139:104076. 10.1016/j.biocontrol.2019.104076

[CR46] Kaczmarek S, Marquardt T, Falenczyk-Kozirog K (2011) Diversity of the Mesostigmata (Acari) in tree-hollows of selected deciduous tree species. Biol Lett 48:29–37

[CR47] Kerezis V, Kiss B, Deucht F, Kontschan J (2019) First record of *Blattisocius mali* (Oudemans, 1929) in Hungary associated with the drosophilid fly *Phortica semivirgo* (Máca, 1977). Redia 102:6972

[CR48] Khodayari S, Fathipour Y, Sedaratian A (2016) Prey stage preference, switching and mutual interference of *Phytoseius plumifer* (Acari: Phytoseiidae) on *Tetranychus urticae* (Acari: Tetranychidae). Syst Appl Acarol 21(3):347–355. 10.11158/saa.21.3.9

[CR49] Kim D, Alvarez M, Lechuga LM, Louis M (2017) Species-specific modulation of food-search behavior by respiration and chemosensation in *Drosophila* larvae. Elife 6:e27057 10.7554/eLife.27057 PMC5584988 28871963

[CR50] Lai YT, Chen JH, Lee LL (2011) Prey selection of a shell-invading leech as predicted by optimal foraging theory with consumption success incorporated into estimation of prey profitability. Func Ecol 25:147–157. 10.1111/j.1365-2435.2010.01774.x

[CR51] Lehtinen PT, Aspi JA (1992) Phytoseiid mite *Paragarmania mali*, associated with drosophilid flies. Acari Physiological and Ecological Aspects of Acari-Host Relationships. European Association of Acarologists: Krynica, Poland

[CR52] Liu S, Li J, Guo K, Qiao H, Xu R, Chen J, Xu C, Chen J (2016) Seasonal phoresy as an overwintering strategy of a phytophagous mite. Sci Rep 6(1):25483. 10.1038/srep25483 PMC4858688 27150196

[CR53] Luong LT, Subasinghe D (2017) A facultative ectoparasite attains higher reproductive success as a parasite than its free-living conspecifics. Exp Appl Acarol 71(1):63–70. 10.1007/s10493-016-0098-2 27858185

[CR54] Luong LT, Brophy T, Stolz E, Chan SJ (2017) State-dependent parasitism by a facultative parasite of fruit flies. Parasitol 144(11):1468–1475. 10.1017/S0031182017000890 28641605

[CR55] Lv J, Yang K, Wang E, Xu X (2016) Prey diet quality affects predation, oviposition and conversion rate of the predatory mite *Neoseiulus barkeri* (Acari: Phytoseiidae). Syst Appl Acarol 21(3):279–287. 10.11158/saa.21.3.3

[CR56] Manly BFJ (1974) A model for certain types of selection experiments. Biometrics 30:281–294. 10.2307/2529649

[CR57] Manu M (2008) Structure and dynamics of the predatory mites (Acari: Mesostigmata- Gamasina) from the central parks and forest ecosystems from/near Bucharest. Species Monitoring in the Central Parks of Bucharest. Ars Docendi, Universitatea Bucureşti

[CR58] Manu M, Szekely L, Oromulu LV, Bărbuceanu D, Honciuc V, Maican S, Fiera C, Purice D, Ion M (2015) Bucharest. In: Kelcey JG (ed) Vertebrates and Invertebrates of European Cities: Selected Non-Avian Fauna. Springer, New York, pp 257–322

[CR59] Manu M, Băncilă RI, Onete M (2018) Importance of moss habitats for mesostigmatid mites (Acari: Mesostigmata) in Romania. Turk J Zool 42(6):673–683. 10.3906/zoo-1712-6

[CR60] Markow TA, O’Grady P (2006) Drosophila: A Guide to Species Identification and Use. Elsevier, London

[CR61] Meehan ML, Caruso T, Lindo Z (2021) Short-term intensive warming shifts predator communities (Parasitiformes: Mesostigmata) in boreal forest soils. Pedobiologia 87:150742. 10.1016/j.pedobi.2021.150742

[CR62] Mendoza JE, Balanza V, Rodríguez-Gómez A, Cifuentes D, Bielza P (2024) Relevance of diet diversification in the coexistence between *Orius laevigatus* and *Amblyseius swirskii*: prey switching and intraguild predation. J Pest Sci 97(4):1993–2005. 10.1007/s10340-024-01762-5

[CR63] Michalska K, Jena MK, Mrowińska A, Nowakowski P, Maciejewska D, Ziółkowska K, Studnicki M, Wit M (2023a) Preliminary studies on the predation of the mite *Blattisocius mali* (Acari: Blattisociidae) on various life stages of spider mite, thrips and fruit fly. Insects 14(9):747. 10.3390/insects14090747 PMC10531691 37754714

[CR64] Michalska K, Mrowińska A, Studnicki M (2023b) Ectoparasitism of the flightless *Drosophila melanogaster* and *D. hydei* by the mite *Blattisocius mali* (Acari: Blattisociidae). Insects 14:146. 10.3390/insects14020146 PMC9961106 36835715

[CR65] Michalska K, Mrowińska A, Studnicki M, Jena MK (2023c) Feeding behaviour of the mite *Blattisocius mali* on eggs of the fruit flies *Drosophila melanogaster* and *D. hydei*. Diversity 15(5):652. 10.3390/d15050652

[CR66] Michalska K, Ibrahim MA, Gwiazdowicz DJ, Magowski W, Laniecki R, Kozłowski MW, Martyka M, Palaczyk A, Studnicki M, Soika G, Mirzwa-Mróz E (2025a) Novel phoretic associations between mites and flies and faunistic composition of phoretic mites at composters in Poland. Euro Zool J 92(1):1052–1064. 10.1080/24750263.2025.2548255

[CR67] Michalska K, Ibrahim MA, Martyka M, Studnicki M, Soika G (2025b) Diversity and abundance of drosophilid fruit flies in compost piles. J Plant Prot Res 65(4):523–534. 10.24425/jppr.2025.155792

[CR68] Michalska K, Jena MK, Studnicki M (2025c) Effect of temperature on functional response of *Blattisocius mali* (Acari: Blattisociidae) preying on the acarid mite *Tyrophagus putrescentiae*. Sci Rep 15(1):15457. 10.1038/s41598-025-00268-z PMC12048524 40316561

[CR69] Mukherjee S, Heithaus MR (2013) Dangerous prey and daring predators: a review. Biol Rev 88(3):550–563 10.1111/brv.12014 23331494

[CR70] Murdoch WW (1969) Switching in general predators: experiments on predator specificity and stability of prey populations. Ecol Mono 39(4):335–354. 10.2307/1942352

[CR71] Murdoch WW, Avery S, Smyth ME (1975) Switching in predatory fish. Ecology 56(5):1094–1105. 10.2307/1936149

[CR72] O’Brien EL, Burger AE, Dawson RD (2005) Foraging decision rules and prey species preferences of northwestern crows (*Corvus caurinus*). Ethology 111(1):77–87. 10.1111/j.1439-0310.2004.01041.x

[CR73] Pekas A, Palevsky E, Sumner JC, Perotti MA, Nesvorna M, Hubert J (2017) Comparison of bacterial microbiota of the predatory mite *Neoseiulus cucumeris* (Acari: Phytoseiidae) and its factitious prey *Tyrophagus putrescentiae* (Acari: Acaridae). Sci Rep 7(1):2. 10.1038/s41598-017-00046-6 PMC5428342 28127053

[CR74] Perez-Leanos A, Loustalot-Laclette MR, Nazario-Yepiz N, Markow TA (2017) Ectoparasitic mites and their *Drosophila* hosts. Fly 11(1):10–18. 10.1080/19336934.2016.1222998 PMC5354228 27540774

[CR75] Perotti A (2001) Prey location and predation rates of predatory mites (Acari: Macrochelidae) on immature stages of pest flies (Diptera: Muscidae). Syst Appl Acarol 6(1):27–33

[CR76] Pirayeshfar F, Safavi SA, Sarraf-Moayeri HR, Messelink GJ (2021) Active and frozen host mite *Tyrophagus putrescentiae* (Acari: Acaridae) influence the mass production of the predatory mite *Blattisocius mali* (Acari: Blattisociidae): life table analysis. Syst Appl Acarol 26(11):2096–2108

[CR77] Polak M (1996) Ectoparasitic Effects on Host Survival and Reproduction: The *Drosophila*–*Macrocheles* Association. Ecology 77(5):1379–1389. 10.2307/2265535

[CR78] Polak M (1998) Effects of ectoparasitism on host condition in the Drosophila–Macrocheles system. Ecology 79(5):1807–1817. 10.1890/0012-9658(1998)079[1807:EOEOHC]2.0.CO;2

[CR79] Porta AO, Soto IM, Soto EM, Saint Esteven A (2020) First record of Macrocheles subbadius (Berlese)(Acari: Macrochelidae) in Argentina, associated with the cactophilic fly Drosophila koepferae Fontdevila & Wasserman (Diptera: Drosophilidae). Rev Soc Entomol Argent 79(4):47–50. 10.25085/rsea.790408

[CR80] Prokopenko CM, Kingdon KA, Dupont DL, Naaykens TM, Prokopenko J, Turner JW, Zabihi-Seissan S, Vander Wal E (2024) Evidence for optimal behavior of predators from parallel field investigations in two distinct wolf-prey systems. 10.1101/2024.06.06.597612. bioRxiv 2024–06

[CR81] Salmane I, Brumelis G (2010) Species list and habitat preference of Mesostigmata mites (Acari, Parasitiformes) in Latvia. Acarologia 50:373–394

[CR82] Schausberger P, Seiter M, Raspotnig G (2020) Innate and learned responses of foraging predatory mites to polar and non-polar fractions of thrips’ chemical cues. Biol Control 151:104371. 10.1016/j.biocontrol.2020.104371

[CR83] Schausberger P, Çekin D, Litin A (2021) Learned predators enhance biological control via organizational upward and trophic top-down cascades. J Appl Ecol 58:158–166. 10.1111/1365-2664.13791 PMC7839590 33536685

[CR84] Schuldiner-Harpaz T, Coll M, Weintraub PG (2016) Prey and pollen food choice depends on previous diet in an omnivorous predatory mite. Environ Entomol 45:995–998. 10.1093/ee/nvw063 27271945

[CR85] Seeman OD, Walter DE (2023) Phoresy and mites: more than just a free ride. Annu Rev Entomol 68:69–88. 10.1146/annurev-ento-120220-013329 36170643

[CR86] Sigsgaard L (2010) Habitat and prey preferences of the two predatory bugs Anthocoris nemorum (L.) and A. nemoralis (Fabricius) (Anthocoridae: Hemiptera-Heteroptera). Biol Control 53:46–54. 10.1016/j.biocontrol.2009.11.005

[CR87] Toft S (1999) Prey choice and spider fitness. J Arachnol 27:301–307

[CR88] Visser M (1981) Prediction of switching and counter-switching based on optimal foraging. Z Tierpsychol 55:129–138. 10.1111/j.1439-0310.1981.tb01264.x

[CR89] Wei J, Liu Y, Sheng F, Wang E, Zhang B, Xu X (2023) Predatory mite *Amblyseius orientalis* prefers egg stage and low density of *Carpoglyphus lactis* prey. Exp Appl Acarol 90:267–276. 10.1007/s10493-023-00805-3 37369863

[CR90] Whitney TD, Sitvarin MI, Roualdes EA, Bonner SJ, Harwood JD (2018) Selectivity underlies the dissociation between seasonal prey availability and prey consumption in a generalist predator. Mol Ecol 27:1739–1748. 10.1111/mec.14554 29543392

[CR92] Yazdanpanah S, Fathipour Y (2025) Exploring the oilseed plant-mediated effects on biological attributes of the predatory mite Neoseiulus cucumeris through its factitious prey Tyrophagus putrescentiae. Bio Sci Techn 35(8):898–908. 10.1080/09583157.2025.2520256

