# Supplementary material for: Predation, prey preference and switching behaviour of the soil predatory mite Blattisocius mali (Mesostigmata: Blattisociidae) on immature stages of Drosophila hydei
Source: Exp Appl Acarol. 2026 Aug 31;97(3):40. doi: 10.1007/s10493-026-01187-y (PMC13529868; doi:10.1007/s10493-026-01187-y)
Supplement: Supplementary file 1 [file 10493_2026_1187_MOESM1_ESM.docx]

The cursory observations on the behaviour and distribution of eggs and larvae of *Drosophila hydei* within a conical cage

**Materials and Methods**

The observations on the distribution of fly eggs, first-instar (L1) and second-instar larvae (L2) within a conical cage were conducted in the presence of mite-reared and fly-reared *B. mali* females, as well as in the absence of predators (control). These observations were performed using similar combinations of relative densities of prey 1 and prey 2 as in the switching experiment: eggs:L1 (40:20 and 20:40), eggs:L2 (40:20 and 20:40), and L1:L2 (40:20 and 20:40). Each treatment combination was replicated five times, and observations were recorded at 2, 6, 12, and 24 hours after eggs and larvae were placed on the bottom of the cage. At each observation interval, the spatial distribution of prey within the cage (bottom surface, cell walls, and underside of the cover slip) was noted. Individuals were considered aggregated when two or more larvae were in direct physical contact or within approximately one body length and treated as dispersed when separated by more than one body length from any other individual. To prevent disturbance, larvae within aggregations were not separated for counting. Thus, counts of individuals within aggregations are approximate, and no statistical analysis was performed.

While checking the distribution of prey in combination with the predator, preliminary behavioural observations were made of fly larvae, both inside and outside the larval aggregations. Several videos were recorded, including behaviour of first- and second-instar larvae within the aggregations, and interactions between larvae (first- and third-instar) and predatory mite while attacking outside larval aggregation. Videos were recorded using a Lucid Vision Labs Triton video camera fitted with a 10 mm lens (TRI023S-CC; Richmond, BC, Canada).

**Results**

**Behaviour of fly larvae in aggregation**

In all examined combinations, with a fly-reared or mite-reared *B. mali* and control, the larvae tended to form aggregations. However, the number of individuals in the aggregation changed very quickly, with some larvae joining, others leaving, remaining separated, or joining a new aggregation. Within a few seconds, groupings of larvae could also disintegrate completely and form anew. Generally, the more larvae were released in the combination, the denser the aggregations formed. While walking, second-instar larvae moved faster than first-instar larvae. However, when aggregated, larvae of these two stages behaved differently. First-instar larvae tended to form a tight tangle of entwining individuals that performed continuous, multidirectional movements (Video S1). In particular, their anterior body parts showed frequent and rapid motion in all directions. Compared with first-instar (L1) larvae, second-instar (L2) larvae created less compact clusters (Video S2). They were generally positioned close side by side and moved forward in the same or opposite direction.

Together with eggs, first-instar larvae were distributed both on the bottom surface alongside eggs and on the cell walls and underside of the cover slip (Table S1). In contrast, second-instar larvae, two hours after the release to the bottom of a cage, all migrated to the cell walls and underside of the cover slip (Table S2). When both larval instars were present within a cage, larval groupings were found all over the cage (Table S3); the aggregations consisted of either first-instar, second-instar or a mix of two larval types, Both first- and second-instar larvae also sporadically transferred eggs (accidentally attached to their bodies) from the bottom to the walls of cages. In a few cases, a reverse process was observed, too, with the displaced eggs returning to the bottom.

**Interaction between predatory mite and fly larvae outside the larval aggregations**

Preliminary observations revealed that all first-, second-, and third-instar larvae of *D. hydei* can vigorously defend themselves against *B. mali* females (Video S3 and Video S4). The defensive responses included the fly’s attempts to escape and struggling with a predator. The predators often attacked the fly larva from its anterior part, near the mouthparts, or from the side, trying to insert their chelicerae into its body. Their interaction may have resembled wrestling: the predatory mite persistently pulled the larva toward it, and the larva countered by pulling the mite in the opposite direction. The larva also often repeatedly vigorously raised its abdomen and waved its body as if trying to shed or detach mites. During combat, *B. mali* females could rotate the larva alternately, once in one direction, once in the opposite direction, effectively limiting its mobility and counteracting its defence. Interactions between *B. mali* females and first- and third-instar larvae of *D. hydei* are illustrated by Video S3 and Video S4, respectively.

Video S1: Aggregation behaviour of first-instar *Drosophila hydei* larvae

The video shows larval behaviour within aggregations 4 hours after the release of *B. mali* female into a cage. Aggregations are present on the cage walls

Video S2: Aggregation behaviour of second-instar *Drosophila hydei* larvae

In this video, larval aggregations are aligned along the upper rim of the cage, on the underside of the cover slip that closes the cage from above. The video was recorded 4 hours after *B. mali* females were released into a cage.

Video S3: Interactions between a *Blattisocius mali* female and a first-instar larva of *Drosophila hydei* outside the larval aggregation

The interaction lasted approximately 1 minute and 15 seconds, culminating with the mite successfully overcoming the first-instar larva’s resistance and beginning to feed. The video illustrates the first minute of this encounter.

Video S4: Interactions between a *Blattisocius mali* female and a third-instar larva of *Drosophila hydei* outside the larval aggregation

The video shows the first minute of the struggle between the third instar larva and the predatory mite. Their interactions continued for approximately five minutes, after which the larva succeeded in detaching the mite entirely from its body.

Table S1. Cursory observations on the distribution of *Drosophila hydei* eggs and first-instar larvae (L1) within a conical cage at 40 eggs:20 L1 or 20 eggs:40 L1 prey ratios, with or without the presence of *Blattisocius mali* females (control), after two, six, twelve and twenty-four hours from the moment of placement of fly eggs and larvae at the cage bottom. Each observation was replicated 5 times. ‘‘+’’ is for the eggs that were spread by larvae on the walls/underside of the cover slip, and ‘‘-’’ is for the eggs transported back to the cage bottom.

| Predator type | Ratio (Eggs: L1) | Replicate | Hours |  | | Bottom | | | | Cell wall and underside of the cover slip | | | | |
| --- | --- | --- | --- | --- | --- | --- | --- | --- | --- | --- | --- | --- | --- | --- |
|  |  |  |  | No. of eggs | No. of larvae | | No. of larvae within aggregations | No. of aggregations | No. of larvae separated | No. of eggs spread (+)/ returned (-) by larvae | No. of larvae | No. of larvae within aggregations | No. of aggregations | No. of larvae separated |
| Mite-reared | 40:20 | 1 | 2 | 40 | 11 | | 4 | 1 | 7 | 0 | 9 | 4 | 1 | 5 |
|  |  |  | 6 | 40 | 11 | | 5 | 1 | 6 | 0 | 8 | 3 | 1 | 5 |
|  |  |  | 12 | 37 | 11 | | 6 | 1 | 5 | +3 | 8 | 0 | 0 | 8 |
|  |  |  | 24 | 38 | 9 | | 3 | 1 | 9 | -1 | 9 | 3 | 1 | 6 |
|  |  | 2 | 2 | 40 | 11 | | 4 | 1 | 7 | 0 | 9 | 5 | 1 | 4 |
|  |  |  | 6 | 40 | 10 | | 3 | 1 | 7 | 0 | 10 | 3 | 1 | 7 |
|  |  |  | 12 | 40 | 15 | | 5; 4 | 2 | 6 | 0 | 5 | 0 | 0 | 5 |
|  |  |  | 24 | 40 | 16 | | 4; 3; 5 | 3 | 4 | 0 | 4 | 0 | 0 | 4 |
|  |  | 3 | 2 | 40 | 9 | | 3 | 1 | 6 | 0 | 11 | 5 | 1 | 6 |
|  |  |  | 6 | 40 | 10 | | 3 | 1 | 7 | 0 | 10 | 4 | 1 | 6 |
|  |  |  | 12 | 40 | 13 | | 5 | 1 | 8 | 0 | 7 | 3 | 1 | 4 |
|  |  |  | 24 | 40 | 12 | | 6 | 1 | 6 | 0 | 7 | 0 | 0 | 7 |
|  |  | 4 | 2 | 40 | 9 | | 5 | 1 | 4 | 0 | 11 | 3 | 1 | 8 |
|  |  |  | 6 | 40 | 11 | | 6 | 1 | 5 | 0 | 9 | 2 | 1 | 7 |
|  |  |  | 12 | 40 | 10 | | 4 | 1 | 6 | 0 | 10 | 3 | 1 | 7 |
|  |  |  | 24 | 40 | 6 | | 2 | 1 | 4 | 0 | 13 | 4 | 1 | 9 |
|  |  | 5 | 2 | 40 | 8 | | 4 | 1 | 4 | 0 | 13 | 5 | 1 | 8 |
|  |  |  | 6 | 40 | 10 | | 3 | 1 | 7 | 0 | 10 | 3 | 1 | 7 |
|  |  |  | 12 | 38 | 9 | | 3 | 1 | 6 | +2 | 10 | 0 | 0 | 10 |
|  |  |  | 24 | 38 | 7 | | 2 | 1 | 5 | 0 | 12 | 5 | 1 | 7 |
|  |  | Mean no. of records/cage |  | 39.55 | 10.4 | | 4.6 | 1.15 | 5.95 | 0.25 | 9.25 | 2.75 | 0.75 | 6.5 |
|  |  |  |  |  |  | |  |  |  |  |  |  |  |  |
| Fly-reared | 40:20 | 1 | 2 | 40 | 13 | | 2; 5 | 2 | 6 | 0 | 7 | 0 | 0 | 7 |
|  |  |  | 6 | 40 | 12 | | 5 | 1 | 7 | 0 | 8 | 4 | 1 | 4 |
|  |  |  | 12 | 40 | 14 | | 8 | 1 | 6 | 0 | 6 | 0 | 0 | 6 |
|  |  |  | 24 | 40 | 16 | | 3; 3; 3 | 3 | 7 | 0 | 3 | 0 | 0 | 3 |
|  |  | 2 | 2 | 40 | 10 | | 4 | 1 | 6 | 0 | 10 | 4 | 1 | 6 |
|  |  |  | 6 | 40 | 11 | | 5 | 1 | 6 | 0 | 9 | 3 | 1 | 6 |
|  |  |  | 12 | 38 | 11 | | 5 | 1 | 6 | +2 | 8 | 4 | 1 | 4 |
|  |  |  | 24 | 39 | 9 | | 3 | 1 | 6 | 0 | 9 | 3 | 1 | 5 |
|  |  | 3 | 2 | 40 | 11 | | 5 | 1 | 6 | 0 | 9 | 0 | 0 | 9 |
|  |  |  | 6 | 40 | 10 | | 5 | 1 | 5 | 0 | 10 | 4 | 1 | 6 |
|  |  |  | 12 | 40 | 12 | | 6 | 1 | 6 | 0 | 8 | 3 | 1 | 5 |
|  |  |  | 24 | 40 | 9 | | 3 | 1 | 6 | 0 | 10 | 4 | 1 | 6 |
|  |  | 4 | 2 | 40 | 9 | | 4 | 1 | 5 | 0 | 11 | 4 | 1 | 7 |
|  |  |  | 6 | 40 | 11 | | 5 | 1 | 6 | 0 | 9 | 3 | 1 | 6 |
|  |  |  | 12 | 40 | 10 | | 5 | 1 | 5 | 0 | 10 | 4 | 1 | 6 |
|  |  |  | 24 | 40 | 7 | | 3 | 1 | 4 | 0 | 12 | 5 | 1 | 7 |
|  |  | 5 | 2 | 40 | 10 | | 2 | 1 | 8 | 0 | 10 | 0 | 0 | 10 |
|  |  |  | 6 | 40 | 10 | | 3 | 1 | 7 | 0 | 10 | 3 | 1 | 7 |
|  |  |  | 12 | 40 | 9 | | 4 | 1 | 5 | 0 | 10 | 4 | 1 | 6 |
|  |  |  | 24 | 39 | 8 | | 4 | 1 | 4 | +1 | 11 | 4 | 1 | 7 |
|  |  | Mean no. of records/cage |  | 39.8 | 10.6 | | 3.95 | 1.15 | 5.85 | 0.15 | 9 | 2.8 | 0.75 | 6.15 |
|  |  |  |  |  |  | |  |  |  |  |  |  |  |  |
| Control | 40:20 | 1 | 2 | 40 | 11 | | 5 | 1 | 6 | 0 | 9 | 0 | 0 | 9 |
|  |  |  | 6 | 40 | 6 | | 0 | 0 | 6 | 0 | 14 | 5 | 1 | 9 |
|  |  |  | 12 | 38 | 8 | | 4 | 1 | 4 | +2 | 12 | 4 | 1 | 8 |
|  |  |  | 24 | 37 | 10 | | 5 | 1 | 5 | +1 | 10 | 3 | 1 | 7 |
|  |  | 2 | 2 | 40 | 5 | | 0 | 0 | 5 | 0 | 15 | 2; 5 | 2 | 8 |
|  |  |  | 6 | 40 | 7 | | 3 | 1 | 4 | 0 | 15 | 5; 3 | 2 | 7 |
|  |  |  | 12 | 40 | 6 | | 0 | 0 | 6 | 0 | 14 | 6 | 1 | 8 |
|  |  |  | 24 | 40 | 8 | | 5 | 1 | 3 | 0 | 12 | 4 | 1 | 8 |
|  |  | 3 | 2 | 40 | 8 | | 3 | 1 | 5 | 0 | 12 | 5 | 1 | 7 |
|  |  |  | 6 | 40 | 7 | | 3 | 1 | 4 | 0 | 13 | 3 | 1 | 10 |
|  |  |  | 12 | 40 | 11 | | 5 | 1 | 6 | 0 | 9 | 0 | 0 | 9 |
|  |  |  | 24 | 36 | 9 | | 3 | 1 | 6 | +4 | 11 | 3 | 1 | 8 |
|  |  | 4 | 2 | 40 | 13 | | 6 | 1 | 7 | 0 | 7 | 0 | 0 | 7 |
|  |  |  | 6 | 40 | 11 | | 6 | 1 | 5 | 0 | 9 | 3 | 1 | 6 |
|  |  |  | 12 | 39 | 15 | | 3; 5 | 2 | 7 | +1 | 5 | 0 | 0 | 5 |
|  |  |  | 24 | 39 | 14 | | 8 | 1 | 6 | 0 | 6 | 0 | 0 | 6 |
|  |  | 5 | 2 | 40 | 11 | | 4 | 1 | 7 | 0 | 9 | 0 | 0 | 9 |
|  |  |  | 6 | 40 | 10 | | 5 | 1 | 5 | 0 | 10 | 4 | 1 | 6 |
|  |  |  | 12 | 40 | 13 | | 6 | 1 | 7 | 0 | 7 | 3 | 1 | 4 |
|  |  |  | 24 | 39 | 14 | | 6 | 1 | 8 | +1 | 6 | 0 | 0 | 6 |
|  |  | Mean no. of records/cage |  | 39.4 | 9.85 | | 4.25 | 0.9 | 5.6 | 2.2 | 10.25 | 2.9 | 0.75 | 7.35 |
|  |  |  |  |  |  | |  |  |  |  |  |  |  |  |
| Mite-reared | 20:40 | 1 | 2 | 20 | 18 | | 4; 12 | 2 | 2 | 0 | 22 | 8; 5 | 2 | 9 |
|  |  |  | 6 | 20 | 20 | | 4; 13 | 2 | 3 | 0 | 19 | 9 | 1 | 10 |
|  |  |  | 12 | 17 | 15 | | 7; 5 | 2 | 3 | +3 | 22 | 11 | 1 | 11 |
|  |  |  | 24 | 17 | 3 | | 0 | 0 | 3 | 0 | 35 | 5; 10 | 2 | 17 |
|  |  | 2 | 2 | 20 | 21 | | 12; 4 | 2 | 5 | 0 | 17 | 9 | 1 | 8 |
|  |  |  | 6 | 19 | 20 | | 12; 4 | 2 | 4 | +1 | 18 | 8 | 1 | 10 |
|  |  |  | 12 | 16 | 20 | | 17 | 1 | 3 | +3 | 20 | 12 | 1 | 8 |
|  |  |  | 24 | 16 | 7 | | 3 | 1 | 4 | 0 | 30 | 5; 5; 7 | 3 | 13 |
|  |  | 3 | 2 | 20 | 21 | | 10; 6 | 2 | 5 | 0 | 19 | 11 | 1 | 8 |
|  |  |  | 6 | 20 | 20 | | 4; 12 | 2 | 4 | 0 | 19 | 12 | 1 | 7 |
|  |  |  | 12 | 20 | 13 | | 3; 7 | 2 | 3 | 0 | 26 | 15 | 1 | 11 |
|  |  |  | 24 | 20 | 0 | | 0 | 0 | 0 | 0 | 39 | 13; 15 | 2 | 11 |
|  |  | 4 | 2 | 20 | 25 | | 11; 10 | 2 | 4 | 0 | 14 | 6 | 1 | 8 |
|  |  |  | 6 | 20 | 20 | | 9; 7 | 2 | 4 | 0 | 19 | 9 | 1 | 10 |
|  |  |  | 12 | 17 | 15 | | 11 | 1 | 4 | +3 | 24 | 12 | 1 | 12 |
|  |  |  | 24 | 17 | 0 | | 0 | 0 | 0 | 0 | 39 | 10; 10 | 2 | 20 |
|  |  | 5 | 2 | 20 | 22 | | 12; 6 | 2 | 4 | 0 | 16 | 7 | 1 | 9 |
|  |  |  | 6 | 20 | 20 | | 10; 6 | 2 | 4 | 0 | 18 | 8 | 1 | 10 |
|  |  |  | 12 | 17 | 22 | | 4; 15 | 2 | 3 | +3 | 16 | 8 | 1 | 10 |
|  |  |  | 24 | 20 | 5 | | 0 | 0 | 5 | -3 | 33 | 5; 7; 5 | 3 | 16 |
|  |  | Mean no. of records/cage |  | 18.8 | 15.35 | | 12 | 1.45 | 3.35 | 0.65 | 23.25 | 12.45 | 1.4 | 10.65 |
|  |  |  |  |  |  | |  |  |  |  |  |  |  |  |
| Fly-reared | 20:40 | 1 | 2 | 20 | 24 | | 3; 7; 9 | 3 | 5 | 0 | 14 | 5 | 1 | 9 |
|  |  |  | 6 | 19 | 20 | | 12 | 1 | 8 | +1 | 18 | 8 | 1 | 10 |
|  |  |  | 12 | 16 | 18 | | 7; 4 | 2 | 7 | +3 | 20 | 12 | 1 | 8 |
|  |  |  | 24 | 16 | 7 | | 3 | 1 | 4 | 0 | 30 | 5; 10 | 2 | 15 |
|  |  | 2 | 2 | 20 | 21 | | 13 | 1 | 8 | 0 | 19 | 8 | 1 | 11 |
|  |  |  | 6 | 20 | 25 | | 4; 4; 5; 4 | 4 | 8 | 0 | 14 | 5 | 1 | 9 |
|  |  |  | 12 | 17 | 15 | | 10 | 1 | 5 | +3 | 23 | 12 | 1 | 12 |
|  |  |  | 24 | 17 | 3 | | 0 | 0 | 3 | 0 | 31 | 5; 10 | 2 | 16 |
|  |  | 3 | 2 | 20 | 23 | | 8; 9 | 2 | 6 | 0 | 17 | 7 | 1 | 10 |
|  |  |  | 6 | 20 | 20 | | 10; 5 | 2 | 5 | 0 | 20 | 10 | 1 | 10 |
|  |  |  | 12 | 20 | 15 | | 11 | 1 | 4 | 0 | 25 | 12 | 1 | 13 |
|  |  |  | 24 | 20 | 0 | | 0 | 0 | 0 | 0 | 38 | 11; 15 | 2 | 12 |
|  |  | 4 | 2 | 20 | 22 | | 13 | 1 | 9 | 0 | 16 | 7 | 1 | 10 |
|  |  |  | 6 | 20 | 20 | | 9; 5 | 2 | 6 | 0 | 18 | 8 | 1 | 10 |
|  |  |  | 12 | 17 | 25 | | 8; 12 | 2 | 5 | +3 | 13 | 4 | 1 | 10 |
|  |  |  | 24 | 20 | 5 | | 0 | 0 | 5 | -3 | 31 | 5; 8; 7 | 3 | 15 |
|  |  | 5 | 2 | 20 | 25 | | 8; 10 | 2 | 7 | 0 | 12 | 3 | 1 | 10 |
|  |  |  | 6 | 20 | 20 | | 8; 6 | 2 | 6 | 0 | 17 | 5 | 1 | 12 |
|  |  |  | 12 | 17 | 15 | | 10 | 1 | 5 | +3 | 20 | 6 | 1 | 14 |
|  |  |  | 24 | 17 | 0 | | 0 | 0 | 0 | 0 | 34 | 15 | 1 | 17 |
|  |  | Mean no. of records/cage |  | 18.8 | 16.15 | | 10.85 | 1.4 | 5.3 | 0.65 | 21.5 | 9.9 | 1.25 | 11.65 |
|  |  |  |  |  |  | |  |  |  |  |  |  |  |  |
| Control | 20:40 | 1 | 2 | 20 | 23 | | 15 | 1 | 10 | 0 | 17 | 5 | 1 | 12 |
|  |  |  | 6 | 20 | 23 | | 15 | 1 | 8 | 0 | 17 | 7 | 1 | 10 |
|  |  |  | 12 | 19 | 20 | | 5; 13 | 2 | 2 | +1 | 20 | 10 | 1 | 10 |
|  |  |  | 24 | 19 | 5 | | 0 | 0 | 5 | 0 | 25 | 12 | 1 | 13 |
|  |  | 2 | 2 | 20 | 23 | | 12 | 1 | 11 | 0 | 17 | 3; 5 | 2 | 9 |
|  |  |  | 6 | 20 | 23 | | 15 | 1 | 8 | 0 | 17 | 7 | 1 | 10 |
|  |  |  | 12 | 20 | 16 | | 11 | 1 | 5 | 0 | 24 | 5; 7 | 2 | 12 |
|  |  |  | 24 | 16 | 9 | | 4 | 1 | 5 | +4 | 31 | 6; 10 | 2 | 15 |
|  |  | 3 | 2 | 20 | 25 | | 7; 12 | 2 | 6 | 0 | 15 | 6 | 1 | 15 |
|  |  |  | 6 | 20 | 20 | | 12 | 1 | 8 | 0 | 20 | 8 | 1 | 12 |
|  |  |  | 12 | 17 | 20 | | 15 | 1 | 5 | +3 | 20 | 4; 6 | 2 | 10 |
|  |  |  | 24 | 12 | 26 | | 10; 10 | 2 | 6 | +5 | 14 | 5 | 1 | 10 |
|  |  | 4 | 2 | 20 | 22 | | 15 | 1 | 8 | 0 | 18 | 7 | 1 | 11 |
|  |  |  | 6 | 20 | 25 | | 5; 10 | 2 | 10 | 0 | 15 | 5 | 1 | 10 |
|  |  |  | 12 | 16 | 15 | | 8 | 1 | 7 | +4 | 25 | 10 | 1 | 15 |
|  |  |  | 24 | 14 | 20 | | 10 | 1 | 10 | +2 | 20 | 8 | 1 | 12 |
|  |  | 5 | 2 | 20 | 20 | | 6; 6 | 2 | 8 | 0 | 20 | 5 | 1 | 15 |
|  |  |  | 6 | 20 | 20 | | 13 | 1 | 7 | 0 | 20 | 7 | 1 | 13 |
|  |  |  | 12 | 20 | 20 | | 7; 5 | 2 | 5 | 0 | 20 | 3; 5 | 2 | 12 |
|  |  |  | 24 | 19 | 20 | | 4; 8 | 2 | 8 | +1 | 20 | 8 | 1 | 12 |
|  |  | Mean no. of records/cage |  | 18.6 | 19.75 | | 12.65 | 1.3 | 7.1 | 1 | 19.75 | 7.7 | 1.25 | 11.9 |

Table S2. Cursory observations on the distribution of *Drosophila hydei* eggs and second-instar larvae (L2) within a conical cage at 40 eggs:20 L2 or 20 eggs:40 L2 prey ratios, with or without the presence of *Blattisocius mali* females (control), after two, six, twelve and twenty-four hours from the moment of placement of fly eggs and larvae at the cage bottom. Each observation was replicated 5 times. ‘‘+’’ is for the eggs that were spread by larvae on the walls/underside of the cover slip, and ‘‘-’’ is for the eggs transported back to the cage bottom.

| Predator type | Ratio (Eggs: L2) | Replicate | Hours |  | | Bottom | | | |  | Cell wall and underside of the cover slip | | | | |
| --- | --- | --- | --- | --- | --- | --- | --- | --- | --- | --- | --- | --- | --- | --- | --- |
|  |  |  |  | No. of eggs | No. of larvae | | No. of larvae within aggregations | No. of aggregations | No. of larvae separated | No. of eggs spread (+)/ returned (-) by larvae | | No. of larvae | No. of larvae within aggregations | No. of aggregations | No. of larvae separated |
| Mite-reared | 40:20 | 1 | 2 | 40 | 0 | | 0 | 0 | 0 | 0 | | 20 | 8 | 1 | 12 |
|  |  |  | 6 | 39 | 5 | | 0 | 0 | 5 | +1 | | 15 | 7 | 1 | 8 |
|  |  |  | 12 | 36 | 0 | | 0 | 0 | 0 | +3 | | 20 | 6; 5 | 2 | 10 |
|  |  |  | 24 | 32 | 7 | | 0 | 0 | 7 | +4 | | 11 | 5 | 1 | 6 |
|  |  | 2 | 2 | 40 | 1 | | 0 | 0 | 1 | 0 | | 18 | 7 | 1 | 11 |
|  |  |  | 6 | 40 | 4 | | 0 | 0 | 4 | 0 | | 15 | 5 | 1 | 10 |
|  |  |  | 12 | 38 | 1 | | 0 | 0 | 1 | +2 | | 18 | 8 | 1 | 10 |
|  |  |  | 24 | 36 | 2 | | 0 | 0 | 2 | +2 | | 17 | 10 | 1 | 9 |
|  |  | 3 | 2 | 40 | 1 | | 0 | 0 | 1 | 0 | | 18 | 6; 4 | 2 | 8 |
|  |  |  | 6 | 40 | 4 | | 0 | 0 | 4 | 0 | | 16 | 3; 7 | 2 | 6 |
|  |  |  | 12 | 32 | 2 | | 0 | 0 | 2 | +8 | | 17 | 4; 5 | 2 | 8 |
|  |  |  | 24 | 25 | 2 | | 0 | 0 | 2 | +7 | | 16 | 6 | 1 | 10 |
|  |  | 4 | 2 | 40 | 4 | | 4 | 1 | 0 | 0 | | 15 | 7 | 1 | 8 |
|  |  |  | 6 | 38 | 5 | | 5 | 0 | 0 | +2 | | 14 | 7 | 1 | 7 |
|  |  |  | 12 | 36 | 3 | | 0 | 0 | 3 | +2 | | 16 | 8 | 1 | 8 |
|  |  |  | 24 | 36 | 0 | | 0 | 0 | 0 | 0 | | 19 | 4; 8 | 2 | 7 |
|  |  | 5 | 2 | 40 | 3 | | 0 | 0 | 3 | 0 | | 17 | 6 | 1 | 11 |
|  |  |  | 6 | 40 | 5 | | 0 | 0 | 5 | 0 | | 14 | 7 | 1 | 7 |
|  |  |  | 12 | 38 | 3 | | 0 | 0 | 3 | +2 | | 16 | 4; 3 | 2 | 9 |
|  |  |  | 24 | 38 | 0 | | 0 | 0 | 0 | 0 | | 19 | 5; 8 | 2 | 6 |
|  |  | Mean no. of records/cage |  | 37.2 | 2.6 | | 0.45 | 0.05 | 2.15 | 1.35 | | 16.55 | 8.15 | 1.35 | 8.55 |
|  |  |  |  |  |  | |  |  |  |  | |  |  |  |  |
| Fly-reared | 40:20 | 1 | 2 | 40 | 0 | | 0 | 0 | 0 | 0 | | 20 | 3; 7 | 2 | 10 |
|  |  |  | 6 | 40 | 5 | | 0 | 0 | 5 | 0 | | 15 | 7 | 1 | 8 |
|  |  |  | 12 | 37 | 0 | | 0 | 0 | 0 | +3 | | 20 | 8 | 1 | 12 |
|  |  |  | 24 | 32 | 10 | | 3 | 1 | 10 | +5 | | 10 | 3 | 1 | 7 |
|  |  | 2 | 2 | 40 | 1 | | 0 | 0 | 1 | 0 | | 18 | 8 | 1 | 10 |
|  |  |  | 6 | 40 | 4 | | 0 | 0 | 4 | 0 | | 15 | 8 | 1 | 7 |
|  |  |  | 12 | 38 | 1 | | 0 | 0 | 1 | +2 | | 18 | 5 | 1 | 13 |
|  |  |  | 24 | 36 | 2 | | 0 | 0 | 2 | +2 | | 17 | 7 | 1 | 10 |
|  |  | 3 | 2 | 40 | 1 | | 0 | 0 | 1 | 0 | | 18 | 7 | 1 | 12 |
|  |  |  | 6 | 40 | 4 | | 2 | 1 | 2 | 0 | | 15 | 7 | 1 | 8 |
|  |  |  | 12 | 31 | 2 | | 0 | 0 | 2 | +9 | | 17 | 3; 5 | 2 | 10 |
|  |  |  | 24 | 25 | 2 | | 0 | 0 | 2 | +6 | | 16 | 5 | 1 | 11 |
|  |  | 4 | 2 | 40 | 0 | | 0 | 0 | 0 | 0 | | 20 | 10 | 1 | 10 |
|  |  |  | 6 | 38 | 5 | | 0 | 0 | 5 | +2 | | 15 | 8 | 1 | 7 |
|  |  |  | 12 | 36 | 3 | | 0 | 0 | 3 | +2 | | 17 | 8 | 1 | 10 |
|  |  |  | 24 | 36 | 0 | | 0 | 0 | 0 | 0 | | 19 | 5; 8 | 2 | 6 |
|  |  | 5 | 2 | 40 | 3 | | 0 | 0 | 3 | 0 | | 17 | 5 | 1 | 12 |
|  |  |  | 6 | 40 | 5 | | 3 | 1 | 2 | 0 | | 15 | 2; 7 | 2 | 6 |
|  |  |  | 12 | 38 | 3 | | 0 | 0 | 3 | +2 | | 17 | 5; 4 | 2 | 8 |
|  |  |  | 24 | 38 | 0 | | 0 | 0 | 0 | 0 | | 20 | 5; 8 | 2 | 7 |
|  |  | Mean no. of records/cage |  | 37.25 | 2.55 | | 0.4 | 0.15 | 2.3 | 1.65 | | 16.95 | 7.9 | 1.3 | 9.2 |
|  |  |  |  |  |  | |  |  |  |  | |  |  |  |  |
| Control | 40:20 | 1 | 2 | 40 | 3 | | 0 | 0 | 3 | 0 | | 17 | 6 | 1 | 11 |
|  |  |  | 6 | 40 | 4 | | 0 | 0 | 4 | 0 | | 16 | 4 | 1 | 12 |
|  |  |  | 12 | 38 | 1 | | 0 | 0 | 1 | +2 | | 19 | 7 | 1 | 12 |
|  |  |  | 24 | 35 | 0 | | 0 | 0 | 0 | +3 | | 20 | 2; 8 | 2 | 10 |
|  |  | 2 | 2 | 40 | 3 | | 0 | 0 | 3 | 0 | | 17 | 5 | 1 | 12 |
|  |  |  | 6 | 40 | 3 | | 0 | 0 | 3 | 0 | | 17 | 7 | 1 | 10 |
|  |  |  | 12 | 37 | 0 | | 0 | 0 | 0 | +3 | | 20 | 5; 6 | 2 | 9 |
|  |  |  | 24 | 37 | 0 | | 0 | 0 | 0 | 0 | | 20 | 5; 7 | 2 | 8 |
|  |  | 3 | 2 | 40 | 3 | | 0 | 0 | 3 | 0 | | 17 | 6 | 1 | 11 |
|  |  |  | 6 | 40 | 4 | | 0 | 0 | 4 | 0 | | 16 | 6 | 1 | 12 |
|  |  |  | 12 | 36 | 2 | | 0 | 0 | 2 | +4 | | 18 | 5; 8 | 2 | 7 |
|  |  |  | 24 | 34 | 0 | | 0 | 0 | 0 | +2 | | 20 | 10 | 1 | 10 |
|  |  | 4 | 2 | 40 | 4 | | 0 | 0 | 4 | 0 | | 16 | 4 | 1 | 12 |
|  |  |  | 6 | 40 | 7 | | 3 | 1 | 4 | 0 | | 13 | 5 | 1 | 8 |
|  |  |  | 12 | 37 | 1 | | 0 | 0 | 1 | +3 | | 19 | 8 | 1 | 12 |
|  |  |  | 24 | 37 | 0 | | 0 | 0 | 0 | 0 | | 20 | 3; 7 | 2 | 10 |
|  |  | 5 | 2 | 40 | 5 | | 3 | 1 | 2 | 0 | | 15 | 5 | 1 | 10 |
|  |  |  | 6 | 40 | 3 | | 2 | 1 | 1 | 0 | | 17 | 5 | 1 | 12 |
|  |  |  | 12 | 38 | 1 | | 0 | 0 | 1 | +2 | | 19 | 8 | 1 | 11 |
|  |  |  | 24 | 35 | 0 | | 0 | 0 | 0 | +3 | | 20 | 12 | 1 | 10 |
|  |  | Mean no. of records/cage |  | 38.2 | 2.2 | | 0.4 | 0.15 | 1.8 | 1.1 | | 17.8 | 7.7 | 1.25 | 10.45 |
|  |  |  |  |  |  | |  |  |  |  | |  |  |  |  |
| Mite-reared | 20:40 | 1 | 2 | 20 | 4 | | 0 | 0 | 4 | 0 | | 36 | 23; 5 | 2 | 8 |
|  |  |  | 6 | 20 | 6 | | 3 | 1 | 3 | 0 | | 34 | 25 | 1 | 10 |
|  |  |  | 12 | 14 | 2 | | 0 | 0 | 2 | +6 | | 38 | 10; 22 | 2 | 6 |
|  |  |  | 24 | 12 | 0 | | 0 | 0 | 0 | +2 | | 39 | 18; 6; 7 | 3 | 8 |
|  |  | 2 | 2 | 20 | 3 | | 0 | 0 | 3 | 0 | | 37 | 16; 13 | 2 | 8 |
|  |  |  | 6 | 20 | 3 | | 0 | 0 | 3 | 0 | | 37 | 23 | 1 | 14 |
|  |  |  | 12 | 20 | 0 | | 0 | 0 | 0 | 0 | | 40 | 9; 25 | 2 | 6 |
|  |  |  | 24 | 16 | 2 | | 2 | 1 | 0 | +4 | | 37 | 6; 15; 4; 5 | 4 | 7 |
|  |  | 3 | 2 | 20 | 25 | | 8; 12 | 2 | 5 | 0 | | 15 | 7 | 1 | 8 |
|  |  |  | 6 | 19 | 20 | | 5; 7 | 2 | 8 | +1 | | 18 | 12 | 1 | 6 |
|  |  |  | 12 | 18 | 2 | | 0 | 0 | 2 | +1 | | 36 | 8; 22 | 2 | 6 |
|  |  |  | 24 | 16 | 0 | | 0 | 0 | 0 | +2 | | 38 | 15; 15 | 2 | 8 |
|  |  | 4 | 2 | 20 | 8 | | 4 | 1 | 4 | 0 | | 32 | 25 | 1 | 8 |
|  |  |  | 6 | 15 | 7 | | 2 | 1 | 5 | +5 | | 33 | 17 | 1 | 16 |
|  |  |  | 12 | 7 | 4 | | 0 | 0 | 4 | +8 | | 36 | 15; 12 | 2 | 9 |
|  |  |  | 24 | 6 | 0 | | 0 | 0 | 0 | +1 | | 39 | 20; 4; 5; 5 | 4 | 6 |
|  |  | 5 | 2 | 20 | 10 | | 3; 3 | 2 | 4 | 0 | | 30 | 22 | 1 | 7 |
|  |  |  | 6 | 14 | 3 | | 3 | 1 | 0 | +6 | | 35 | 22 | 1 | 13 |
|  |  |  | 12 | 5 | 0 | | 0 | 0 | 0 | +9 | | 38 | 10; 25 | 2 | 3 |
|  |  |  | 24 | 5 | 0 | | 0 | 0 | 0 | 0 | | 38 | 13; 12; 6 | 3 | 7 |
|  |  | Mean no. of records/cage |  | 15.35 | 4.95 | | 2.6 | 0.55 | 2.35 | 2.25 | | 34.3 | 26.2 | 1.9 | 8.2 |
| Fly-reared | 20:40 | 1 | 2 | 18 | 4 | | 0 | 0 | 4 | +2 | | 36 | 23 | 1 | 13 |
|  |  |  | 6 | 18 | 6 | | 2 | 1 | 4 | 0 | | 34 | 18; 7 | 2 | 9 |
|  |  |  | 12 | 17 | 2 | | 0 | 0 | 2 | +1 | | 38 | 15; 7 | 2 | 6 |
|  |  |  | 24 | 9 | 7 | | 0 | 0 | 7 | +7 | | 33 | 17; 4; 4 | 3 | 7 |
|  |  | 2 | 2 | 20 | 4 | | 0 | 0 | 4 | 0 | | 36 | 16; 6; 6 | 3 | 9 |
|  |  |  | 6 | 20 | 3 | | 0 | 0 | 3 | 0 | | 37 | 17; 8 | 2 | 11 |
|  |  |  | 12 | 20 | 0 | | 0 | 0 | 0 | 0 | | 40 | 10; 25 | 2 | 5 |
|  |  |  | 24 | 16 | 2 | | 0 | 0 | 2 | +4 | | 38 | 12; 10; 5; 5 | 4 | 6 |
|  |  | 3 | 2 | 20 | 10 | | 5 | 1 | 5 | 0 | | 30 | 23 | 1 | 7 |
|  |  |  | 6 | 19 | 10 | | 6 | 1 | 4 | +1 | | 28 | 22 | 1 | 7 |
|  |  |  | 12 | 18 | 2 | | 0 | 0 | 2 | +1 | | 36 | 11; 20 | 2 | 5 |
|  |  |  | 24 | 16 | 0 | | 0 | 0 | 0 | +2 | | 38 | 16; 15 | 2 | 8 |
|  |  | 4 | 2 | 20 | 8 | | 3 | 1 | 5 | 0 | | 32 | 22 | 1 | 10 |
|  |  |  | 6 | 15 | 7 | | 4 | 1 | 3 | +5 | | 33 | 25 | 1 | 8 |
|  |  |  | 12 | 7 | 4 | | 0 | 0 | 4 | +8 | | 36 | 18; 10 | 2 | 8 |
|  |  |  | 24 | 4 | 0 | | 0 | 0 | 0 | +3 | | 39 | 23 | 1 | 16 |
|  |  | 5 | 2 | 20 | 10 | | 3 | 1 | 7 | 0 | | 29 | 18 | 1 | 11 |
|  |  |  | 6 | 14 | 3 | | 0 | 0 | 3 | +6 | | 34 | 25 | 1 | 9 |
|  |  |  | 12 | 5 | 0 | | 0 | 0 | 0 | +9 | | 37 | 7; 25 | 2 | 5 |
|  |  |  | 24 | 4 | 0 | | 0 | 0 | 0 | +1 | | 37 | 15; 8; 4 | 3 | 10 |
|  |  | Mean no. of records/cage |  | 15 | 4.1 | | 1.15 | 0.3 | 2.95 | 2.55 | | 35.05 | 26.05 | 1.85 | 8.5 |
|  |  |  |  |  |  | |  |  |  |  | |  |  |  |  |
| Control | 20:40 | 1 | 2 | 20 | 7 | | 3 | 1 | 4 | 0 | | 33 | 20 | 1 | 12 |
|  |  |  | 6 | 20 | 25 | | 5; 15 | 2 | 4 | 0 | | 15 | 11 | 1 | 4 |
|  |  |  | 12 | 15 | 6 | | 0 | 0 | 6 | +5 | | 34 | 21 | 1 | 13 |
|  |  |  | 24 | 15 | 0 | | 0 | 0 | 0 | 0 | | 40 | 18; 5; 5; 5 | 4 | 7 |
|  |  | 2 | 2 | 20 | 10 | | 7 | 1 | 3 | 0 | | 30 | 23 | 1 | 7 |
|  |  |  | 6 | 19 | 9 | | 4 | 1 | 5 | +1 | | 31 | 20 | 1 | 13 |
|  |  |  | 12 | 20 | 2 | | 0 | 0 | 2 | -1 | | 38 | 8; 12; 10 | 3 | 8 |
|  |  |  | 24 | 20 | 0 | | 0 | 0 | 0 | 0 | | 40 | 15; 16 | 2 | 10 |
|  |  | 3 | 2 | 20 | 10 | | 5 | 1 | 5 | 0 | | 30 | 25 | 1 | 7 |
|  |  |  | 6 | 20 | 15 | | 4; 5 | 2 | 6 | 0 | | 35 | 23 | 1 | 12 |
|  |  |  | 12 | 16 | 0 | | 0 | 0 | 0 | +4 | | 40 | 28 | 1 | 13 |
|  |  |  | 24 | 16 | 2 | | 0 | 0 | 2 | 0 | | 38 | 27 | 1 | 13 |
|  |  | 4 | 2 | 20 | 6 | | 3 | 1 | 3 | 0 | | 34 | 23 | 1 | 11 |
|  |  |  | 6 | 19 | 16 | | 5; 7 | 2 | 4 | +1 | | 24 | 15 | 1 | 9 |
|  |  |  | 12 | 17 | 3 | | 3 | 1 | 0 | +2 | | 37 | 12; 10; 10 | 3 | 5 |
|  |  |  | 24 | 17 | 0 | | 0 | 0 | 0 | 0 | | 40 | 21; 11 | 2 | 8 |
|  |  | 5 | 2 | 20 | 12 | | 5 | 1 | 7 | 0 | | 38 | 25 | 1 | 13 |
|  |  |  | 6 | 20 | 13 | | 7 | 1 | 6 | 0 | | 27 | 20 | 1 | 7 |
|  |  |  | 12 | 17 | 1 | | 0 | 0 | 1 | +3 | | 39 | 27 | 1 | 12 |
|  |  |  | 24 | 17 | 0 | | 0 | 0 | 0 | 0 | | 40 | 30 | 1 | 10 |
|  |  | Mean no. of records/cage |  | 18.4 | 6.85 | | 3.9 | 0.7 | 2.9 | 0 | | 34.15 | 24.8 | 1.45 | 9.7 |

Table S3. Cursory observations on the distribution of *Drosophila hydei* first-instar (L1) and second-instar larvae (L2) within a conical cage at 40 L1:20 L2 or 20 L1:40 L2 prey ratios, with or without the presence of *Blattisocius mali* females (control), after two, six, twelve and twenty-four hours from the moment of placement of fly eggs and larvae at the cage bottom. Each observation was replicated 5 times.

| Predator type | Ratio (L1: L2) | Replicate | Hours |  | | Bottom | | | | | Cell wall and underside of the cover slip | | | | | |
| --- | --- | --- | --- | --- | --- | --- | --- | --- | --- | --- | --- | --- | --- | --- | --- | --- |
|  |  |  |  | No. of L1 | No. of L2 | | No. of larvae within aggregations | No. of aggregations | No. of larvae separated | | No. of L1 | No. of L2 | No. of larvae within aggregations | No. of aggregations | No. of larvae separated | |
|  |  |  |  |  |  |  |  |  | L1 | L2 |  |  |  |  | L1 | L2 |
| Mite-reared | 40: 20 | 1 | 2 | 3 | 5 | | 6 | 1 | 0 | 2 | 33 | 14 | 12; 4; 8; 8; 8 | 5 | 6 | 1 |
|  |  |  | 6 | 3 | 0 | | 3 | 1 | 0 | 0 | 30 | 19 | 19; 8; 8; 8 | 4 | 6 | 0 |
|  |  |  | 12 | 0 | 0 | | 0 | 0 | 0 | 0 | 32 | 19 | 12; 12; 12; 9 | 4 | 8 | 0 |
|  |  |  | 24 | 0 | 0 | | 0 | 0 | 0 | 0 | 32 | 16 | 12; 15; 11 | 3 | 8 | 2 |
|  |  | 2 | 2 | 20 | 10 | | 5; 7; 6; 7 | 4 | 3 | 2 | 13 | 10 | 4 | 1 | 5 | 1 |
|  |  |  | 6 | 2 | 0 | | 0 | 0 | 2 | 0 | 31 | 20 | 12; 10; 10; 11 | 4 | 7 | 1 |
|  |  |  | 12 | 4 | 6 | | 2; 8 | 2 | 0 | 0 | 27 | 10 | 10; 10; 7 | 3 | 8 | 2 |
|  |  |  | 24 | 9 | 8 | | 6; 8 | 2 | 2 | 1 | 19 | 4 | 11 | 1 | 10 | 2 |
|  |  | 3 | 2 | 10 | 5 | | 5; 6 | 2 | 3 | 1 | 23 | 14 | 9; 15 | 2 | 8 | 5 |
|  |  |  | 6 | 4 | 4 | | 6 | 1 | 2 | 2 | 29 | 15 | 14; 12; 8 | 3 | 9 | 1 |
|  |  |  | 12 | 0 | 0 | | 0 | 0 | 0 | 0 | 33 | 19 | 5; 14; 14 | 3 | 9 | 0 |
|  |  |  | 24 | 4 | 0 | | 4 | 1 | 0 | 0 | 27 | 15 | 16; 8; 8; 5 | 4 | 5 | 0 |
|  |  | 4 | 2 | 12 | 6 | | 6; 5; 5 | 3 | 2 | 0 | 21 | 13 | 15; 5; 6 | 3 | 8 | 0 |
|  |  |  | 6 | 3 | 4 | | 4 | 1 | 2 | 1 | 28 | 13 | 15; 4; 4; 7 | 4 | 9 | 2 |
|  |  |  | 12 | 0 | 0 | | 0 | 0 | 0 | 0 | 30 | 16 | 10; 11; 15 | 3 | 7 | 3 |
|  |  |  | 24 | 6 | 1 | | 5 | 1 | 2 | 0 | 24 | 12 | 10; 8; 8 | 3 | 3 | 7 |
|  |  | 5 | 2 | 12 | 12 | | 5; 17 | 2 | 2 | 0 | 20 | 8 | 5; 15 | 2 | 6 | 2 |
|  |  |  | 6 | 4 | 5 | | 5; 4 | 2 | 0 | 0 | 27 | 13 | 15; 10; 6 | 3 | 6 | 3 |
|  |  |  | 12 | 1 | 0 | | 0 | 0 | 2 | 0 | 30 | 15 | 12; 5; 6; 6; 6 | 5 | 8 | 2 |
|  |  |  | 24 | 7 | 3 | | 7 | 1 | 2 | 1 | 22 | 11 | 12; 13 | 2 | 9 | 0 |
|  |  | Mean no. of records/cage |  | 5.2 | 3.45 | | 7.1 | 1.2 | 1.2 | 0.5 | 26.55 | 13.8 | 30.4 | 3.1 | 7.25 | 1.7 |
|  |  |  |  |  |  | |  |  |  |  |  |  |  |  |  |  |
| Fly-reared | 40: 20 | 1 | 2 | 4 | 5 | | 9 | 1 | 0 | 0 | 30 | 14 | 13; 8; 8; 8 | 4 | 7 | 0 |
|  |  |  | 6 | 2 | 0 | | 2 | 1 | 0 | 0 | 30 | 19 | 7; 8; 12; 7; 8 | 5 | 6 | 1 |
|  |  |  | 12 | 0 | 0 | | 0 | 0 | 0 | 0 | 32 | 19 | 10; 12; 8; 7; 8 | 5 | 6 | 0 |
|  |  |  | 24 | 0 | 0 | | 0 | 0 | 0 | 0 | 27 | 17 | 15; 12; 10 | 3 | 6 | 1 |
|  |  | 2 | 2 | 22 | 7 | | 12; 6; 6 | 3 | 2 | 3 | 11 | 13 | 10; 6 | 2 | 7 | 1 |
|  |  |  | 6 | 6 | 0 | | 6 | 1 | 0 | 0 | 27 | 20 | 24; 15 | 2 | 7 | 1 |
|  |  |  | 12 | 5 | 6 | | 7; 4 | 2 | 0 | 0 | 26 | 12 | 10; 10; 11 | 3 | 7 | 0 |
|  |  |  | 24 | 9 | 12 | | 6; 8 | 2 | 4 | 3 | 31 | 4 | 15; 15 | 2 | 4 | 1 |
|  |  | 3 | 2 | 10 | 9 | | 4; 10 | 2 | 2 | 3 | 23 | 11 | 16; 11 | 2 | 5 | 2 |
|  |  |  | 6 | 6 | 5 | | 7; 4 | 2 | 0 | 0 | 23 | 15 | 20; 6; 5 | 3 | 5 | 2 |
|  |  |  | 12 | 3 | 0 | | 3 | 1 | 0 | 0 | 23 | 18 | 15; 10; 10 | 3 | 5 | 1 |
|  |  |  | 24 | 7 | 4 | | 7; 4 | 2 | 0 | 0 | 15 | 12 | 14; 13 | 2 | 0 | 0 |
|  |  | 4 | 2 | 12 | 6 | | 6; 12 | 2 | 0 | 0 | 21 | 13 | 12; 12; 4 | 3 | 5 | 1 |
|  |  |  | 6 | 3 | 4 | | 7 | 1 | 0 | 0 | 28 | 15 | 20; 5; 5; 5 | 4 | 8 | 0 |
|  |  |  | 12 | 0 | 0 | | 0 | 0 | 0 | 0 | 28 | 17 | 23; 15 | 2 | 6 | 1 |
|  |  |  | 24 | 6 | 1 | | 7 | 1 | 0 | 0 | 20 | 15 | 14; 7; 7 | 3 | 5 | 2 |
|  |  | 5 | 2 | 22 | 12 | | 7; 10; 10 | 3 | 4 | 3 | 22 | 8 | 10; 12 | 2 | 6 | 2 |
|  |  |  | 6 | 6 | 5 | | 7; 4 | 2 | 0 | 0 | 22 | 15 | 10; 6; 6; 4; 4 | 5 | 5 | 2 |
|  |  |  | 12 | 2 | 0 | | 2 | 1 | 0 | 0 | 24 | 20 | 25; 6; 6 | 3 | 7 | 0 |
|  |  |  | 24 | 2 | 3 | | 5 | 1 | 0 | 0 | 21 | 15 | 10; 15; 3 | 3 | 7 | 1 |
|  |  | Mean no. of records/cage |  | 6.35 | 3.95 | | 9.1 | 1.4 | 0.6 | 0.6 | 24.2 | 14.6 | 32.15 | 3.05 | 5.7 | 0.95 |
|  |  |  |  |  |  | |  |  |  |  |  |  |  |  |  |  |
| Control | 40: 20 | 1 | 2 | 7 | 3 | | 4 | 1 | 4 | 2 | 33 | 17 | 24; 5; 5; 7 | 4 | 7 | 1 |
|  |  |  | 6 | 4 | 5 | | 5 | 1 | 3 | 1 | 36 | 15 | 15; 16; 6; 6 | 4 | 8 | 0 |
|  |  |  | 12 | 4 | 0 | | 0 | 0 | 2 | 2 | 36 | 20 | 12; 16; 9; 5; 5; 5 | 6 | 3 | 1 |
|  |  |  | 24 | 5 | 2 | | 3 | 1 | 3 | 1 | 35 | 18 | 16; 16; 8 | 3 | 12 | 1 |
|  |  | 2 | 2 | 7 | 3 | | 0 | 0 | 3 | 0 | 33 | 17 | 15; 15; 8 | 3 | 11 | 1 |
|  |  |  | 6 | 6 | 0 | | 0 | 0 | 0 | 0 | 34 | 20 | 15; 15; 5; 15 | 4 | 3 | 1 |
|  |  |  | 12 | 3 | 0 | | 0 | 0 | 0 | 0 | 37 | 20 | 18; 19; 11 | 3 | 6 | 1 |
|  |  |  | 24 | 5 | 3 | | 0 | 0 | 3 | 0 | 35 | 17 | 15; 15; 5; 7 | 4 | 8 | 2 |
|  |  | 3 | 2 | 6 | 3 | | 0 | 0 | 2 | 1 | 34 | 17 | 20; 10; 16 | 3 | 5 | 0 |
|  |  |  | 6 | 4 | 3 | | 0 | 0 | 0 | 3 | 36 | 17 | 15; 18; 11 | 3 | 5 | 2 |
|  |  |  | 12 | 4 | 2 | | 0 | 0 | 2 | 0 | 36 | 18 | 20; 10; 10 | 3 | 13 | 1 |
|  |  |  | 24 | 5 | 2 | | 0 | 0 | 2 | 0 | 35 | 18 | 16; 16; 3; 13 | 4 | 5 | 0 |
|  |  | 4 | 2 | 6 | 4 | | 7 | 1 | 0 | 3 | 34 | 16 | 18; 10; 12; 4 | 4 | 4 | 2 |
|  |  |  | 6 | 18 | 5 | | 5,11 | 2 | 4 | 3 | 22 | 15 | 11; 12; 10 | 3 | 4 | 0 |
|  |  |  | 12 | 18 | 7 | | 6,11 | 2 | 5 | 3 | 22 | 13 | 15; 15 | 2 | 5 | 0 |
|  |  |  | 24 | 20 | 13 | | 16,12 | 2 | 2 | 3 | 20 | 7 | 13; 9 | 2 | 5 | 0 |
|  |  | 5 | 2 | 7 | 3 | | 3 | 1 | 4 | 3 | 33 | 17 | 12; 16; 13; 4 | 4 | 4 | 1 |
|  |  |  | 6 | 5 | 2 | | 0 | 0 | 4 | 3 | 35 | 18 | 15; 15; 17 | 3 | 5 | 1 |
|  |  |  | 12 | 6 | 0 | | 6 | 1 | 0 | 0 | 34 | 20 | 12; 13; 15; 6 | 4 | 7 | 1 |
|  |  |  | 24 | 1 | 3 | | 0 | 0 | 3 | 1 | 39 | 17 | 12; 18; 8; 5; 5; 5 | 6 | 5 | 1 |
|  |  | Mean no. of records/cage |  | 7.05 | 3.15 | | 4.45 | 0.6 | 2.3 | 1.45 | 32.95 | 16.85 | 42.6 | 3.6 | 6.25 | 0.85 |
|  |  |  |  |  |  | |  |  |  |  |  |  |  |  |  |  |
| Mite-reared | 20:40 | 1 | 2 | 3 | 5 | | 4 | 1 | 2 | 2 | 11 | 35 | 15; 17; 7 | 3 | 2 | 5 |
|  |  |  | 6 | 3 | 3 | | 3 | 1 | 2 | 1 | 10 | 38 | 14; 16; 11 | 3 | 1 | 6 |
|  |  |  | 12 | 1 | 0 | | 1 | 1 | 0 | 0 | 12 | 38 | 14; 10; 10; 10 | 4 | 0 | 6 |
|  |  |  | 24 | 0 | 0 | | 0 | 0 | 0 | 0 | 13 | 35 | 13; 10; 10; 10 | 4 | 1 | 4 |
|  |  | 2 | 2 | 4 | 3 | | 3 | 1 | 3 | 1 | 10 | 34 | 7; 15; 7; 7 | 4 | 0 | 8 |
|  |  |  | 6 | 4 | 2 | | 3 | 1 | 1 | 2 | 10 | 35 | 22; 17 | 2 | 1 | 5 |
|  |  |  | 12 | 0 | 0 | | 0 | 0 | 0 | 0 | 14 | 37 | 12; 14; 10; 10 | 4 | 1 | 4 |
|  |  |  | 24 | 0 | 0 | | 0 | 0 | 0 | 0 | 13 | 34 | 10; 22; 5; 5 | 4 | 1 | 6 |
|  |  | 3 | 2 | 3 | 5 | | 4 | 1 | 3 | 1 | 11 | 35 | 8; 12; 18 | 3 | 1 | 7 |
|  |  |  | 6 | 2 | 3 | | 2 | 1 | 2 | 1 | 12 | 37 | 18; 9; 9; 9 | 4 | 0 | 5 |
|  |  |  | 12 | 0 | 0 | | 0 | 0 | 0 | 0 | 14 | 38 | 12; 14; 10; 10 | 4 | 1 | 5 |
|  |  |  | 24 | 4 | 3 | | 3 | 1 | 1 | 3 | 9 | 35 | 12; 10; 18 | 3 | 3 | 1 |
|  |  | 4 | 2 | 10 | 2 | | 2; 10 | 2 | 0 | 0 | 3 | 36 | 18; 10; 6 | 3 | 2 | 5 |
|  |  |  | 6 | 2 | 3 | | 3 | 1 | 1 | 1 | 11 | 32 | 15; 10; 8; 8 | 4 | 3 | 0 |
|  |  |  | 12 | 0 | 0 | | 0 | 0 | 0 | 0 | 13 | 34 | 9; 10; 12; 12 | 4 | 2 | 3 |
|  |  |  | 24 | 0 | 0 | | 0 | 0 | 0 | 0 | 13 | 33 | 7; 13; 20 | 3 | 1 | 5 |
|  |  | 5 | 2 | 5 | 4 | | 4 | 1 | 2 | 3 | 11 | 33 | 12; 8; 8; 8 | 4 | 2 | 5 |
|  |  |  | 6 | 3 | 3 | | 2 | 1 | 3 | 1 | 12 | 34 | 10; 14; 12; 6 | 4 | 0 | 4 |
|  |  |  | 12 | 0 | 0 | | 0 | 0 | 0 | 0 | 14 | 34 | 9; 9; 12; 12 | 4 | 2 | 5 |
|  |  |  | 24 | 0 | 0 | | 0 | 0 | 0 | 0 | 13 | 31 | 21; 18 | 2 | 2 | 3 |
|  |  | Mean no. of records/cage |  | 2.2 | 1.8 | | 2.2 | 0.65 | 1 | 0.8 | 11.45 | 34.9 | 40.8 | 3.5 | 1.3 | 4.6 |
|  |  |  |  |  |  | |  |  |  |  |  |  |  |  |  |  |
| Fly-reared | 20:40 | 1 | 2 | 3 | 6 | | 5 | 1 | 3 | 1 | 10 | 31 | 12; 12; 14 | 3 | 3 | 1 |
|  |  |  | 6 | 3 | 1 | | 4 | 1 | 0 | 0 | 9 | 33 | 17; 10; 10 | 3 | 2 | 3 |
|  |  |  | 12 | 0 | 0 | | 0 | 0 | 0 | 0 | 12 | 33 | 9; 9; 12; 12 | 4 | 0 | 3 |
|  |  |  | 24 | 0 | 0 | | 0 | 0 | 0 | 0 | 10 | 32 | 16; 8; 8; 8 | 4 | 2 | 2 |
|  |  | 2 | 2 | 4 | 5 | | 5 | 1 | 3 | 2 | 10 | 30 | 10; 16; 5; 5 | 4 | 3 | 2 |
|  |  |  | 6 | 4 | 4 | | 5 | 1 | 2 | 1 | 7 | 28 | 10; 15; 5 | 3 | 3 | 2 |
|  |  |  | 12 | 0 | 0 | | 0 | 0 | 0 | 0 | 10 | 31 | 17; 10; 10 | 3 | 2 | 2 |
|  |  |  | 24 | 0 | 0 | | 0 | 0 | 0 | 0 | 7 | 27 | 2; 10; 5; 5 | 4 | 1 | 2 |
|  |  | 3 | 2 | 3 | 5 | | 3 | 1 | 3 | 2 | 11 | 30 | 10; 12; 7; 7 | 4 | 4 | 2 |
|  |  |  | 6 | 0 | 0 | | 0 | 0 | 0 | 0 | 11 | 32 | 12; 9; 9; 9 | 4 | 3 | 2 |
|  |  |  | 12 | 0 | 0 | | 0 | 0 | 0 | 0 | 8 | 30 | 12; 10; 10 | 3 | 2 | 4 |
|  |  |  | 24 | 0 | 0 | | 0 | 0 | 0 | 0 | 6 | 27 | 11; 18 | 2 | 2 | 2 |
|  |  | 4 | 2 | 4 | 4 | | 6 | 1 | 2 | 0 | 3 | 28 | 6; 7; 10; 6 | 4 | 1 | 2 |
|  |  |  | 6 | 0 | 1 | | 0 | 0 | 0 | 0 | 10 | 29 | 19; 8; 8 | 3 | 0 | 6 |
|  |  |  | 12 | 0 | 0 | | 0 | 0 | 0 | 0 | 7 | 27 | 6; 12; 12 | 3 | 1 | 6 |
|  |  |  | 24 | 0 | 0 | | 0 | 0 | 0 | 0 | 5 | 27 | 10; 20 | 2 | 2 | 2 |
|  |  | 5 | 2 | 5 | 5 | | 5 | 1 | 3 | 2 | 8 | 29 | 17; 8; 8 | 3 | 3 | 2 |
|  |  |  | 6 | 1 | 0 | | 1 | 1 | 0 | 0 | 8 | 29 | 12; 15; 6 | 3 | 2 | 4 |
|  |  |  | 12 | 0 | 0 | | 0 | 0 | 0 | 0 | 9 | 29 | 14; 10; 10 | 3 | 2 | 2 |
|  |  |  | 24 | 0 | 0 | | 0 | 0 | 0 | 0 | 9 | 27 | 14; 18 | 2 | 1 | 2 |
|  |  | Mean no. of records/cage |  | 1.35 | 1.55 | | 1.7 | 0.4 | 0.8 | 0.4 | 8.5 | 29.45 | 33.7 | 3.2 | 1.95 | 2.65 |
|  |  |  |  |  |  | |  |  |  |  |  |  |  |  |  |  |
| Control | 20:40 | 1 | 2 | 5 | 6 | | 6 | 1 | 2 | 3 | 15 | 34 | 14; 16; 7; 7 | 4 | 2 | 3 |
|  |  |  | 6 | 4 | 5 | | 5 | 1 | 3 | 1 | 16 | 35 | 16; 14; 15 | 3 | 2 | 4 |
|  |  |  | 12 | 2 | 3 | | 3 | 1 | 2 | 0 | 18 | 37 | 15; 16; 10; 10 | 4 | 2 | 2 |
|  |  |  | 24 | 0 | 0 | | 0 | 0 | 0 | 0 | 20 | 40 | 14; 16; 10; 10; 10 | 5 | 2 | 3 |
|  |  | 2 | 2 | 6 | 3 | | 6 | 1 | 3 | 0 | 14 | 37 | 14; 17; 5; 5; 5 | 5 | 2 | 3 |
|  |  |  | 6 | 4 | 0 | | 4 | 1 | 0 | 0 | 16 | 40 | 13; 13; 5; 22 | 4 | 3 | 2 |
|  |  |  | 12 | 0 | 0 | | 0 | 0 | 0 | 0 | 0 | 40 | 15; 19; 12; 4; 4 | 5 | 2 | 4 |
|  |  |  | 24 | 0 | 0 | | 0 | 0 | 0 | 0 | 20 | 40 | 24; 20; 5; 5 | 4 | 1 | 7 |
|  |  | 3 | 2 | 5 | 3 | | 5 | 1 | 0 | 3 | 15 | 37 | 19; 6; 4; 15 | 4 | 0 | 6 |
|  |  |  | 6 | 2 | 1 | | 3 | 1 | 0 | 0 | 18 | 39 | 7; 10; 17; 10; 10 | 5 | 1 | 3 |
|  |  |  | 12 | 0 | 0 | | 0 | 0 | 0 | 0 | 20 | 40 | 17; 9; 9; 10; 10 | 5 | 3 | 0 |
|  |  |  | 24 | 0 | 0 | | 0 | 0 | 0 | 0 | 20 | 40 | 14; 15; 25 | 3 | 3 | 23 |
|  |  | 4 | 2 | 10 | 7 | | 6,6 | 2 | 2 | 3 | 10 | 33 | 10; 12; 8; 8 | 4 | 2 | 3 |
|  |  |  | 6 | 7 | 5 | | 6 | 1 | 3 | 3 | 13 | 35 | 8; 12; 12; 6; 6 | 5 | 3 | 23 |
|  |  |  | 12 | 3 | 3 | | 3 | 1 | 2 | 1 | 17 | 37 | 14; 14; 12; 12 | 4 | 1 | 3 |
|  |  |  | 24 | 0 | 0 | | 0 | 0 | 0 | 0 | 20 | 40 | 17; 10; 10; 20 | 4 | 3 | 0 |
|  |  | 5 | 2 | 10 | 7 | | 4,13 | 2 | 0 | 0 | 10 | 33 | 24; 8; 6 | 3 | 3 | 23 |
|  |  |  | 6 | 7 | 5 | | 4,8 | 2 | 0 | 0 | 13 | 35 | 8; 8; 9; 8; 8 | 5 | 1 | 6 |
|  |  |  | 12 | 0 | 0 | | 0 | 0 | 0 | 0 | 20 | 40 | 15; 10; 10; 12; 12 | 5 | 2 | 1 |
|  |  |  | 24 | 0 | 0 | | 0 | 0 | 0 | 0 | 20 | 40 | 19; 10; 20; 5 | 4 | 0 | 6 |
|  |  | Mean no. of records/cage |  | 3.25 | 2.4 | | 4.1 | 0.75 | 0.85 | 0.7 | 15.75 | 37.6 | 49.85 | 4.25 | 1.9 | 6.25 |
